# Landmark‐guided region‐based spatial normalization for functional magnetic resonance imaging

**DOI:** 10.1002/hbm.25865

**Published:** 2022-04-12

**Authors:** Hengda He, Qolamreza R. Razlighi

**Affiliations:** ^1^ Department of Biomedical Engineering Columbia University New York New York USA; ^2^ Department of Radiology Weill Cornell Medicine New York New York USA

**Keywords:** functional MRI, MRI, registration, spatial normalization

## Abstract

As the size of the neuroimaging cohorts being increased to address key questions in the field of cognitive neuroscience, cognitive aging, and neurodegenerative diseases, the accuracy of the spatial normalization as an essential preprocessing step becomes extremely important. Existing spatial normalization methods have poor accuracy particularly when dealing with the highly convoluted human cerebral cortex and when brain morphology is severely altered (e.g., aging populations). To address this shortcoming, we propose a novel spatial normalization technique that takes advantage of the existing surface‐based human brain parcellation to automatically identify and match regional landmarks. To simplify the nonlinear whole brain registration, the identified landmarks of each region and its counterpart are registered independently with topology‐preserving deformation. Next, the regional warping fields are combined by an inverse distance weighted interpolation technique to have a global warping field for the whole brain. To ensure that the final warping field is topology‐preserving, we used simultaneously forward and reverse maps with certain symmetric constraints to yield bijectivity. We have evaluated our proposed solution using both simulated and real (structural and functional) human brain images. Our evaluation shows that our solution can enhance structural correspondence compared to the existing methods. Such improvement also increases the sensitivity and specificity of the functional imaging studies, reducing the required number of subjects and subsequent study costs. We conclude that our proposed solution can effectively substitute existing substandard spatial normalization methods to deal with the demand of large cohorts which is now common in clinical and aging studies.

## Graphical abstract

We propose a novel spatial normalization solution for neuroimaging data particularly functional magnetic resonance imaging (fMRI), which takes advantage of existing surface‐based parcellation technique to perform automatic landmark detection and matching for subsequent region‐based volumetric registration. The regional non‐linear warping fields obtained independently for each brain region are combined, to generate a single global warping field and enforce topology‐preserving properties. We showed that our solution improves the structural correspondence of brain regions in comparison to the top‐preforming existing methods, and also improves the sensitivity and specificity of the fMRI activation at the group‐level activation statistics.

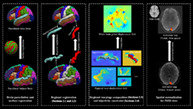

## 1 INTRODUCTION

Spatial normalization is an essential preprocessing step in many neuroimaging studies that makes between‐subjects and between‐groups comparisons possible by warping each subject's brain image onto a common or standard space. Spatial normalization is often performed by an underlying subject‐to‐subject or subject‐to‐standard image registration. Image registration, by definition, serves to make all subjects' neuroanatomical regions correspond to a standard space and consequently to each other. Without neuroanatomical correspondences, it is challenging if not impossible, to perform any across‐subjects univariate or multivariate statistical analyses (likely the most essential step in obtaining and interpreting scientific results from neuroimaging data). Yet inter‐subject registration of the brain, especially the human brain cerebral cortex, remains challenging due to its highly convoluted patterns of sulci and gyri with large inter‐subject morphological variability. For example, cortical folding (e.g., sulci branches) is not consistent between subjects in many cortical regions (Van Essen, 2005). This not only makes their registration challenging, but also increases the likelihood of false‐positive findings in neuroimaging studies (Desai et al., 2005; Liu et al., 2017). Better correspondence between neuroanatomical regions will improve the statistical power to detect any brain effect and will increase spatial specificity, resulting in a reduced number of required subjects and consequently study costs (Miller et al., 2005).

The most commonly employed spatial normalization methods perform either a volume‐based nonlinear registration of structural images in 3D Euclidean space, or a surface‐based nonlinear registration of the cerebral cortex surfaces in 2D parametric surface space. For example, currently widely‐used volume‐based brain image registration methods include large deformation diffeomorphic metric mapping (LDDMM) (Beg et al., 2005; Zhang et al., 2017), advanced normalization tools (ANTs) (Avants et al., 2008), Quicksilver (Yang et al., 2017), and VoxelMorph (Balakrishnan et al., 2019). Alternatively, commonly used surface‐based methods include FreeSurfer (Fischl et al., 1999) and spherical demons (Yeo et al., 2010). There are also volume and surface hybrid methods available, that extend the cortex correspondence specified using surface‐based registration to 3D Euclidean space. For example, Joshi et al. and Lepore et al. used harmonic mapping, whereas Postelnicu et al. used Navier operator of elastic diffusion for extending the surface correspondence to the volumetric space (Guo et al., 2005; Joshi et al., 2007; Lepore et al., 2010; Postelnicu et al., 2009).

The following lists the major concerns of volume‐based spatial normalization: (1) Human neuroanatomical regions are distributed throughout a highly folded surface of the cerebral cortex which often makes the Euclidean distance less reliable in segregating adjacent regions with completely distinct functionality. For instance, the posterior and anterior banks of the *Sylvian fissure* have completely distinct functionalities, but they could be seen adjacent to each other in the Euclidean space. Therefore, even a small misalignment in the Euclidean space can exert drastic consequences in matching neuroanatomical regions. (2) There is almost no difference in the intensity of the cerebral gray‐matter throughout the entire cortex. This makes intensity‐based similarity measures, used often as the cost function in image registration, less sensitive in distinguishing different neuroanatomical regions throughout the cortex, particularly in adjacent ones. As we have demonstrated in our simulation, even if volume‐based registration was able to align the cortical folding patterns between‐subjects, it would still be less likely to correspond perfectly between different cortical regions along the aligned cortical gray‐matter ribbons. (3) Every nonlinear image registration method relies on an underlying optimization step. Due to the complexity and large inter‐subject variability in the cortical morphology of human brain, the optimization objective function becomes nonconvex almost at all cases causing many local optima that could be detected as the final solution, without providing an accurate correspondence between the regions. Thus, even the best performing volume‐based nonlinear registration (ANTs) results in a poor correspondence between the cortical regions (Dice similarity coefficient (DSC) of 0.6–0.7 (Klein et al., 2009)). Therefore, it is not surprising that even the recently‐developed deep learning methods still have comparable performance as ANTs (Balakrishnan et al., 2019; Yang et al., 2017). Such poor correspondence can attribute the location of brain activation to different regions of the standard space in group‐level analysis, reducing the statistical power available to detect significant effects.

To address these shortcomings, surface‐based methods were proposed to directly align brain folding patterns of gyri and sulci based on their underlying curvature instead of relying on voxel intensity. For instance, Fischl et al. (2008) demonstrated that compared to nonlinear volumetric methods, a surface‐based method more consistently aligns brain *cyto‐architectonic* boundaries (Brodmann areas). Optimization in surface‐based methods is more efficient as it works in a 2D surface space with fewer degrees of freedom. However, all surface‐based spatial normalization methods are required to project functional data extracted from gray‐matter volume to a cortical surface. This mapping process is challenging in practice and potentially problematic. For example, cortical surfaces are typically extracted from structural scans and projected onto functional image space. Due to excess geometric distortion in fast acquisition techniques, such as echo planar imaging (EPI) often used for functional magnetic resonance imaging (fMRI) acquisition and their low resolution, co‐registration between functional and structural scans is likely to have inaccuracies that directly result in sampling nongray‐matter regions. By sampling nongray‐matter regions or regions from a neighboring gyrus/sulcus onto the cortical surface, functional activation can easily get lost and affect the results of the group‐level analysis. It has been shown that during mapping of functional activation from the volume to the surface, the functional signal can be diluted to neighboring gyri. This effect can be consistent across subjects and detected at the group‐level, resulting in a false‐positive cluster of brain activation otherwise absent in volume‐based spatial normalization (Tucholka et al., 2012). Another shortcoming of the surface‐based methods is that they cannot be applied for registering sub‐cortical regions. Finally, almost all widely used brain image registration techniques whether volume‐ or surface‐based, or a hybrid method, are based on solving the optimization problem of matching the whole brain at once, while suffering from the local minimum problem, resulting in poor correspondence between brain cortical regions.

To alleviate the local minimum problem in volume, surface, or hybrid methods, we use a region‐based local registration technique (Razlighi, 2016), in which each brain's cortical/sub‐cortical region is independently registered to its corresponding region. The superiority of the region‐based method is due to the fact that inter‐subject variability in each brain region is much smaller than the inter‐subject variability in their whole brains. We have evaluated this automatic landmark‐guided region‐based local registration technique, which can accurately match brain cortical segmented regions with an averaged DSC of 0.8 (He & Razlighi, 2020). However, in our previous method and some other region of interest (ROI) based methods (Miller et al., 2005), applying regional warping fields individually to each region will result in overlaps and gaps between regions after the warping, which makes it challenging to warp whole brain images and functional activation maps covering multiple regions. To overcome this, in this article, we propose to combine the regional locally estimated nonlinear displacement warping fields to obtain a smooth whole brain global displacement field using an inverse distance weighted (IDW) interpolation. However, the interpolated global deformation field is not guaranteed to preserve the topology during warping. Thus, we propose a residual compensation iterative algorithm to enforce bijectivity and topology‐preserving properties into the global deformation field, which is also applicable to other given nontopology‐preserving deformation fields. During the residual compensation regularization, to avoid losing the match of brain regions, we applied a region‐based demons registration to match the cerebral cortex mask and sub‐cortical regions at the same time.

The present study proposes a novel region‐based volumetric spatial normalization method, which is the first study using regionally and independently estimated local nonlinear displacement fields to composite a global bijective displacement field for the whole brain. And it is also the first study to use dense brain tissue surface vertices as pseudo‐landmarks to guide the volumetric registration. Compared to volumetric methods, instead of matching voxel intensity, we propose to use landmarks guidance as the registration similarity measurement. In our method, we directly estimate a volumetric warping field using corresponding vertices on the surface of WM/GM (WM: white matter; GW: gray matter) and GM/CSF (CSF: cerebrospinal fluid) boundaries as automatically extracted landmarks in the 3D Euclidean space, which allows us to incorporate brain anatomical information and features into the volumetric registration process with exact correspondence. Compared to the surface‐based methods, as our method extend the surface‐based registration results to the 3D Euclidean space, our solution circumvents the projection of volumetric fMRI data onto the cortical surface. And our method is applicable to not only the cerebral cortex but also sub‐cortical, cerebellar, ventricular, and other brain regions. In summary, we propose an automatic algorithm to extract and match the corresponding vertices on the WM/GM and GM/CSF boundary surfaces in each anatomical region; Next, to estimate a topology preserving warping field, we independently estimate a landmark‐guided volumetric warping field via large deformation diffeomorphisms.

This article is organized as follows: We first explain the detail of the proposed landmark‐guided region‐based spatial normalization (LG‐RBSN) method in Section 2. We also explain the subjects' demographics and acquisition parameters used to acquire MRI scans in our experiments. In Section 3, we first used simulated 2D images to illustrate problems associated with volume‐based methods as well as demonstrating the effectiveness of our proposed method in registering these simulated special cases. We then compare our method with other nonlinear whole brain registration methods using real human brain images. Our results show that our method achieves higher DSC than the existing top performing volumetric registration method (ANTs; Klein et al., 2009) and a hybrid registration method (combined volumetric and surface registration, CVS; Postelnicu et al., 2009) in warping the brain's cortical regions, sub‐cortical regions, and cerebral WM. In experiments with fMRI spatial normalization, our method performs better than ANTs and CVS with regard to the specificity and sensitivity of the fMRI activation at the group‐level activation statistics. Finally, we include a discussion in Section 4 and we conclude the article in Section 5.

## 2 MATERIALS AND METHODS

**FIGURE 1:**
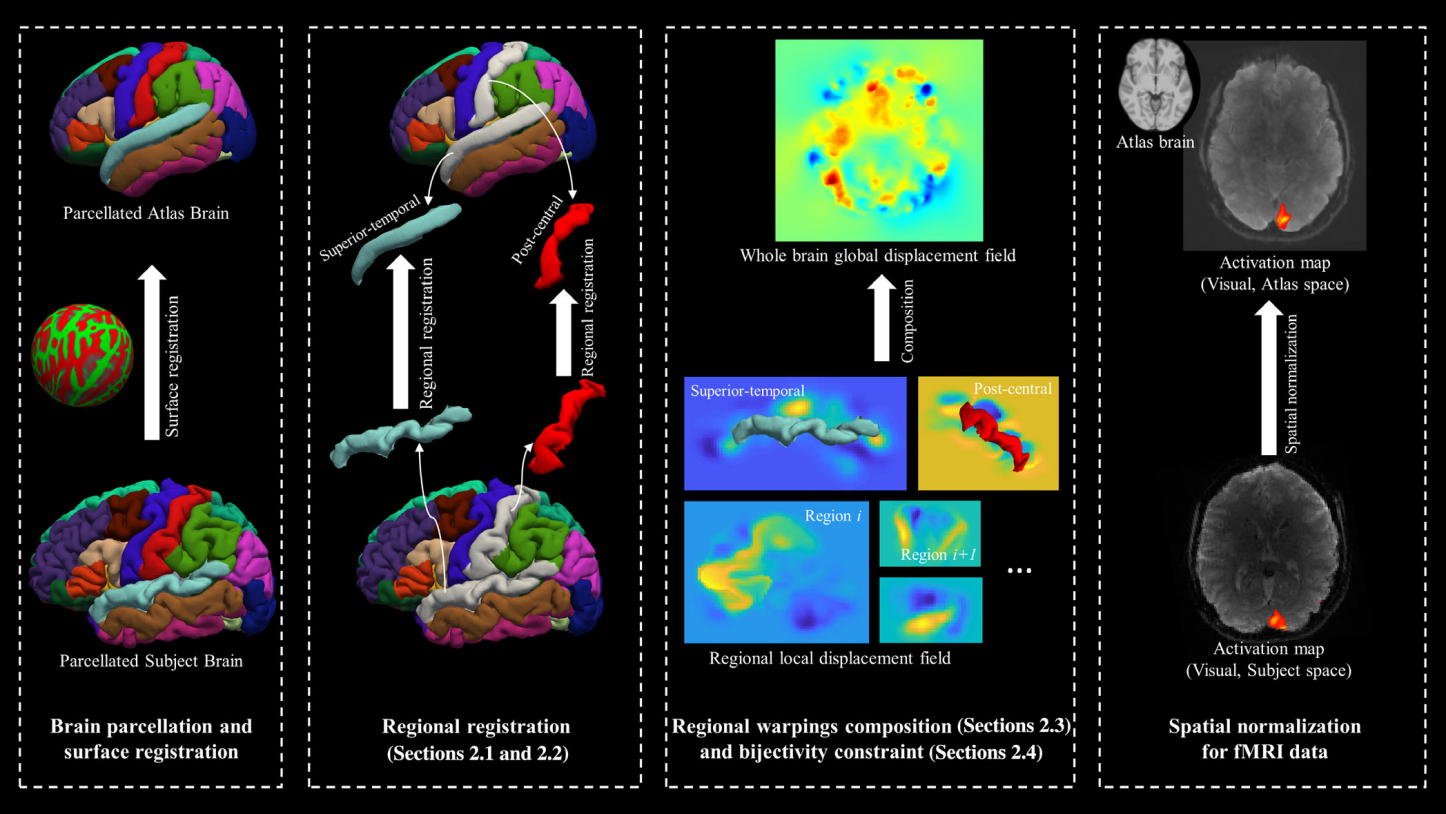
The pipeline for the landmark‐guided region‐based spatial normalization (LG‐RBSN) solution. Subject's T1 image is processed with FreeSurfer for surface reconstruction and parcellation. Then, an automatic regional landmark extraction and matching approach (Section 2.1) is used to extract regional landmarks from the results of surface registration. For each region independently, the landmark‐guided large deformation diffeomorphic registration (Section 2.2) is performed resulting in a distinct displacement field for that region. We then combine the regional displacement fields together using a novel interpolation technique (Section 2.3) to give a single global displacement field for the whole brain. Finally, a residual compensation approach is used to enforce bijectivity property into the global deformation field (Section 2.4)

In this section, we describe the details of our novel spatial normalization solution, specifically focused on accurately matching brain cortical regions, which have complicated topological variations. Constituting 40% of total brain mass, cortical regions are of primary interest to neuroscience field as the information‐processing brain tissue (Saladin & McFarland, 2008). Figure 1 illustrates a flowchart of different processes required for our proposed LG‐RBSN solution which starts with a surface reconstruction and parcellation of the cerebral cortex followed by our automatic regional landmark extraction and matching approach. Then, the landmark‐guided geodesic shooting large deformation diffeomorphic registration is performed independently for each region resulting in a distinct warping field for that region. We then combine the regional warping field together using a novel interpolation technique, IDW, to give a single global warping field for the whole brain. Finally, the forward and reverse warping fields residual compensation is used to enforce bijectivity property into the global deformation field with a region‐based demons registration to keep matching of the sub‐cortical regions and cerebral cortex during regularization.

### 2.1 Automatic regional landmark extraction and matching

Landmark‐based image registration can be an alternative to the volumetric registration that circumvents the use of intensity‐based similarity measures to estimate a volumetric warping field. Whereas existing landmark‐based image registrations generate a direct and accurate correspondence between images and generally do not face the local minimum problem, they require a manual identification of corresponding landmarks, which is labor intensive, subject to human error, and usually has a limited number of landmarks. For example, Joshi et al. used manually labeled sulci features as landmarks to guide the registration (Joshi et al., 2005, 2007). Durrleman et al. (2008) used manually delineated sulcal lines, and represented them as currents in the registration. Joshi et al. (2012) also used manually delineated sulci, but instead of currents they introduced a velocity representation of the sulci curves. Auzias et al. (2011) applied an automatic technique to extract, identify, and simplify sulcal landmarks, and the sulcal edges were represented as mathematical measures in the registration. Here, instead of using landmarks only from sulci lines, we define landmarks as dense pseudo‐landmarks from the vertices of the brain tissue surface triangular meshes to guide the registration, which covers both the sulci and gyri of the cortical surface and the white matter surface. And we propose an automatic landmark identifying and matching procedure that accounts for approximately 2000 landmarks per region, with totally around 136,000 landmarks per subject.

**FIGURE 2:**
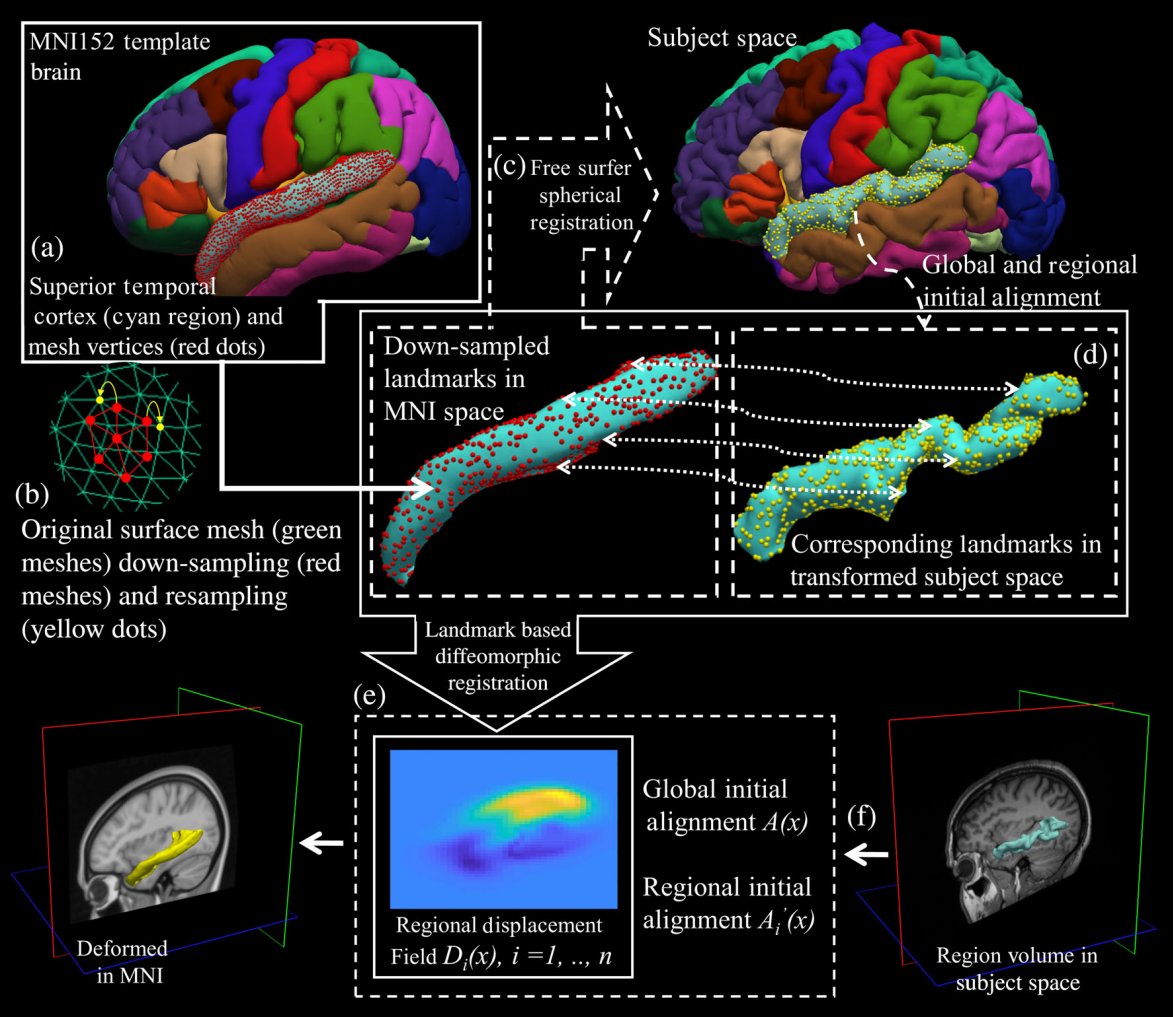
Illustrate our method for automatic landmark extraction and matching for landmark‐based regional nonlinear registration with example on superior temporal cortex (STC) region. In step (a), for STC region (cyan color), vertices of the GW/WM and GM/CSF boundaries triangular meshes are extracted as landmarks (GM/CSF surface vertices shown as red dots, GW/WM surface vertices are not showing in the figure); In step (b), landmarks of STC region are down‐sampled by down‐sampling the original dense surface mesh (green meshes) to a sparser surface mesh (red meshes) and sampled back to the original vertices (yellow dots) to keep consistency; In step (c), correspondence of STC regional landmarks between the MNI template and the subject is established through spherical registration (corresponding landmarks in subject space shown as yellow dots); In step (d), corresponding regional landmarks are initially aligned with linear transformations in 3D Euclidean space; In step (e), a diffeomorphic nonlinear landmark‐based registration is used to generate regional warping field for STC region. The step (f) is only showing that STC regional warping field can be used to warp the subject's regional volume onto MNI template space. CSF, cerebrospinal fluid; GW, gray matter; WM: white matter

Our method starts with processing subjects' structural T1‐weighted images and MNI152 template using FreeSurfer pipeline (http://surfer.nmr.mgh.harvard.edu/, RRID:SCR_001847), resulting in 68 cortical regions (Fischl et al., 2004). However, using FreeSurfer for initial reconstruction and delineation of the human cerebral cortex is just an arbitrary choice, other accurate surface reconstruction and delineation methods can also be used for this purpose. The framework of landmarks matching and regional registration is illustrated in Figure 2. For each region, vertices of the WM surface (boundary between GM and WM) and pial surface (boundary between GM and CSF) triangular meshes are extracted as landmarks using the labels assigned by FreeSurfer's cortical surface parcellation algorithm. The pial surface landmarks of one of the cortical regions (superior temporal cortex) are illustrated in Figure 2a. To reduce computation, the landmarks for regions with large number of landmarks are down‐sampled. Based on the time‐accuracy tradeoff, a fixed down‐sampling rate is assigned to each region varying from 10% to 60%. In our experiment, the down‐sampling process only sacrifices a small amount of accuracy, but it substantially reduces the computation time. Down‐sampled vertices are then matched back to the closest vertices on the original sphere mesh to maintain consistency between down‐sampled vertices and original ones. The down‐sampled pial surface landmarks of one of the cortical regions (superior temporal cortex) are illustrated in Figure 2c. Next, the correspondence of regional landmarks between each subject and the MNI template is established through the FreeSurfer spherical registration algorithm (Fischl et al., 1999). Specifically, the MNI vertices of each region are transformed into the subject's spherical surface space using the spherical registration. For each projected vertex, the closest vertex of the subject's original vertices is identified as the matching landmark in the subject's space.

We then performed two initial linear alignments between corresponding landmarks for each region, including a global and a local transformation, independently. First, assuming that we have m regions $R_{i}\;\left(i=1,\ldots ,m\right)$, subject space $S\left(x\right),x\in \Omega_{S}\subset {\mathbb{R}}^{3}$, and MNI space $M\left(x\right),x\in \Omega_{M}\subset {\mathbb{R}}^{3}$, the subject's brain structural image is registered to the MNI brain template entirely with an affine transformation $A\left(x\right)$, and subsequently each subject's cortical region mask is separately registered to its corresponding region in MNI space with a translation only transformation $A_{i}^{\prime }\left(x\right),i=1,\ldots ,m$. In our experiments, translation only transformations generate more appropriate linear initial alignments for brain cortical regions without causing any over‐fitting for the subsequent regional nonlinear registration. Regional landmarks are transformed using these two linear transformations for initial alignment. The final regional warping field is the concatenation of two initial linear transformations and the nonlinear warping field.

### 2.2 Landmark‐based large deformation diffeomorphic registration via geodesic shooting

Traditional landmark‐based nonlinear image registration methods are based on smoothing spline interpolation with different radial basis functions, such as thin‐plate spline (Bookstein, 1989). Yet due to their questionable invertibility, spline‐based methods often fail when dealing with the human brain's highly convoluted topology, especially in cases that require large deformation, dense placement, or curved trajectories of landmarks (Joshi & Miller, 2000). To address the problem of large deformation, we use a large diffeomorphic deformation method with geodesic shooting (Vaillant et al., 2004) to estimate a valid warping field for each cortical region. With the optimal landmarks' geodesic paths, both forward and reverse diffeomorphic regional deformation maps are estimated. Here, we provide a review of the large diffeomorphic deformation registration for the sake of completeness. For the initially aligned corresponding landmarks $x_{n}$ and $y_{n}\;(n$ in 1,…,N) in the same coordinate, image warping is described as a time‐varying flow quantified by a transport equation $\frac{\mathrm{d\phi}\left(x,t\right)}{\mathrm{dt}}=v\left(\phi \left(x,t\right),t\right)$. With time denoted by $t\in \left[0,1\right]$, spatial space by $x\in \Omega \subset {\mathbb{R}}^{3}$, a time‐dependent flow by $\phi \left(x,t\right)$, and a velocity vector field by $v\left(x,t\right)$. This ordinary differential equation has the solution of $\hat{\phi }\left(x,t\right)=\int_{0}^{t}\hat{v}\left(\hat{\phi }\left(x,t\prime \right),t\prime \right)\mathrm{dt}\prime +x$, with initial condition $\phi \left(x,0\right)=x$. The final deformation map is taken as the end point of this image warping flow which is $\hat{\phi }\left(x,1\right)$, and the final displacement vector field is denoted as $D_{i}\left(x\right)={\hat{\phi }}_{i}\left(x,1\right)-x$ for the $i^{\mathrm{th}}$ region.

The deformation map is constrained to be diffeomorphism through a regularization penalty on the smoothness of the velocity vector field. Thus, to obtain diffeomorphic registration, the optimization objective function becomes the following:

(1) $$J\left(v\right)=\int_{0}^{1}\int_{\Omega }{\left\|\mathrm{Lv}\left(x,t\right)\right\|}^{2}\text{dxdt}+\sum_{n=1}^{N}{\left[y_{n}-\phi \left(x_{n},1\right)\right]}^{T}\sum_{n}^{-1}\left[y_{n}-\phi \left(x_{n},1\right)\right]$$

where $x_{n}$ and $y_{n}$ are moving and fixed landmarks, respectively, and L is the linear momentum operator defined on a Hilbert space V with $\mathrm{Lv}\;\left(v\in V\right)$ considered as a mapping from V to ${\mathbb{R}}^{3}$, which satisfies ${\left\|v_{t}\right\|}_{V}^{2}=Lv_{t}\left(v_{t}\right)$. The mapping $Lv_{t}$ is defined as the momentum of the system at time t. The second term in the objective function is the Mahalanobis distance between transformed moving landmarks and fixed landmarks, and $\sum_{n}$ is the covariance matrix of landmarks, quantifying the error of inexact matching of landmarks (as soft correspondences between landmarks).

Under geodesic shooting settings, the geodesic path of landmarks can be represented with an initial momentum space at the initial time point and an initial configuration of moving landmarks. Considering the conservation law of system momentum, the objective function becomes a function of the initial momentum. The optimization problem can be solved with typical gradient descent, and the optimal landmarks geodesic path is uniquely specified using the Hamiltonian principle with the optimal initial momentum and the moving landmarks configuration. The velocity vector field is assumed as Gaussian random fields and is interpolated over the entire domain. Finally, the displacement vector field $D_{i}\left(x\right),x\in \Omega_{S}$ is obtained for each region $R_{i}\;\left(i=1,\ldots ,m\right)$ with the solution of transport equation at t=1. By interpolating the reverse velocity vector field from the reverse of the optimal landmarks geodesic path using the same scheme, we obtain a reverse displacement vector field $D_{i}^{-1}\left(x\right),x\in \Omega_{M}$. We use an available Matlab script for landmark‐based diffeomorphic image registration to perform our landmark‐guided nonlinear regional registration (Sommer et al., 2011).

### 2.3 IDW interpolation of neighboring region‐based displacement composition

**FIGURE 3:**
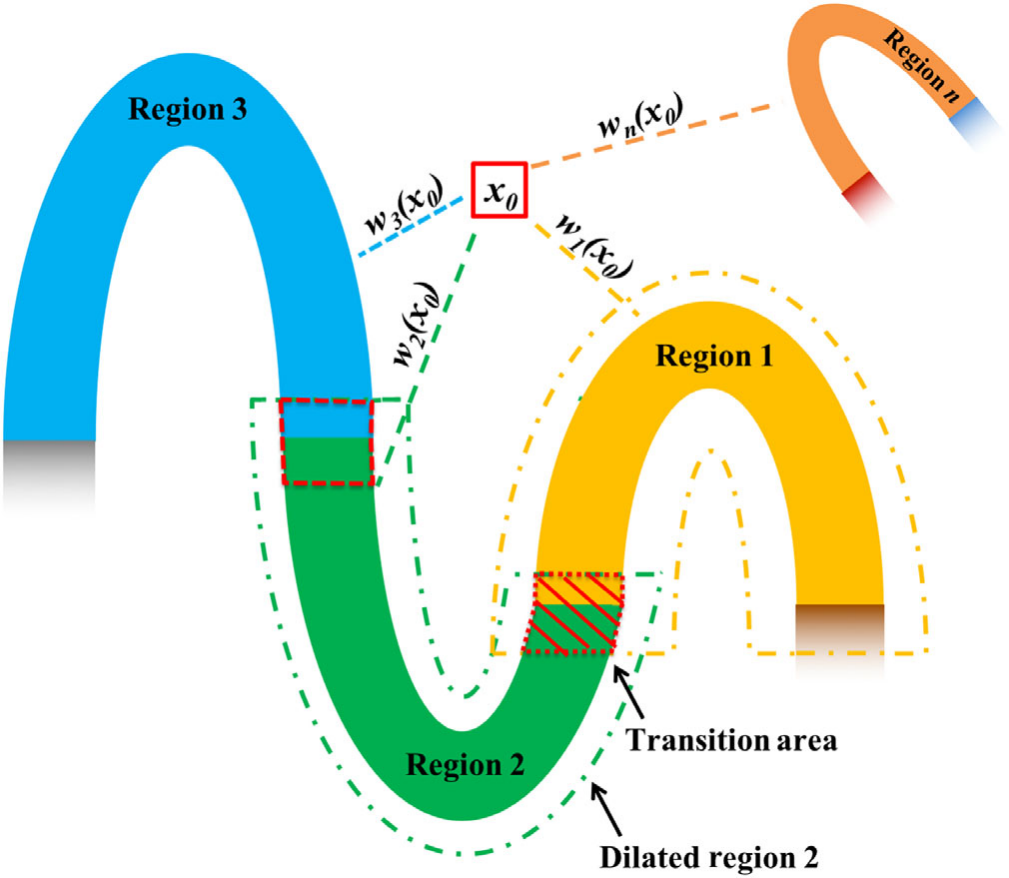
Combining adjacent regions' nonlinear displacement fields using inverse distance weighted interpolation. For each location in the background and region‐to‐region transition area, the displacement is calculated as a normalized weighted average of the displacement values in all regional warping fields at that location. The weight is based on the inverse of the closest distance between the location and the region. *W_i_* (*x*) is the normalized weight at location *x* for region *i*

To estimate a single smooth global warping field that is applicable to all brain regions and can be applied to warp the whole brain all together at once, we propose the IDW interpolation method to combine all regional warping fields of the cortical regions. Regional displacement fields composition is illustrated in Figure 3. First, for region i, the global initial alignment $A\left(x\right)$, regional initial alignment $A_{i}^{\prime }\left(x\right)$, and nonlinear regional displacement vector field $D_{i}\left(x\right)$ are concatenated to a single regional displacement vector field $T_{i}\left(x\right)=D_{i}\left(x\right)\circ A_{i}^{\prime }\left(x\right)\circ A\left(x\right)$. For interpolation between regions, morphology operations are performed to identify the region‐to‐region transition area in the brain. For example, for region i denoted as $R_{i}$, all other regions $R_{j}$ j=1,…,m,j≠i are unioned (${⋃}_{j}R_{j}$) then dilated (${⋃}_{j}R_{j}(⨁\mathrm{SE}$) to intersect with the dilated region i ($R_{i}⨁\mathrm{SE}$) for finding the transition area of the region i that is $\left[R_{i}⨁\mathrm{SE}\right]\cap \left[\left({⋃}_{j}R_{j}\right)⨁\mathrm{SE}\right]$, which leads to region i without transition area denoted as $R_{i}^{*}=R_{i}-\left[R_{i}⨁\mathrm{SE}\right]\cap \left[\left({⋃}_{j}R_{j}\right)⨁\mathrm{SE}\right],j=1,\ldots ,m,j\neq i$, where SE is the structural element. Here, we use a sphere with a radius of two voxels as our SE. The shortest distance from spatial location x to $R_{i}^{*}$, which is called $d_{i}\left(x\right)$, is used as the weighting factor in our IDW interpolation. Given subject space $S\left(x\right),x\in \Omega_{S}\subset {\mathbb{R}}^{3}$ and MNI space $M\left(x\right),x\in \Omega_{M}\subset {\mathbb{R}}^{3}$, the global forward displacement vector field $u_{\mathrm{SM}}\left(x\right)$ is a normalized weight of each regional displacement vector field,

(2) $$u_{\mathrm{SM}}\left(x\right)=\sum_{i=1}^{m}w_{i}\left(x\right)T_{i}\left(x\right)$$

where $w_{i}\left(x\right)$ is the normalized weight defined as follows:

(3) $$w_{i}\left(x\right)=\left\{\begin{array}{c} 1,\;x\in R_{i}^{*}\\ 0,\;x\in R_{j}^{*},j=1,\ldots ,m,j\neq i\\ \frac{q_{i}\left(x\right)}{\sum_{j=1}^{m}q_{j}\left(x\right)},\text{otherwise} \end{array}\right.$$

Here, $q_{i}\left(x\right)=\frac{1}{d_{i}{\left(x\right)}^{\mu }}$ and $\sum_{i=1}^{m}w_{i}\left(x\right)=1$. The first derivative of the interpolated global forward displacement vector field $u_{\mathrm{SM}}\left(x\right)$ is continuous with μ>1 (Shepard, 1968), and in our method we set μ=4. The same IDW interpolation is also applied to the reverse regional displacement vector fields $T_{i}^{-1}\left(x\right)={\left[A\left(x\right)\right]}^{-1}\circ {\left[A_{i}^{\prime }\left(x\right)\right]}^{-1}\circ D_{i}^{-1}\left(x\right)$ to obtain the global reverse displacement $u_{\mathrm{MS}}\left(x\right)$. With the IDW interpolated displacement vector fields $u_{\mathrm{SM}}\left(x\right)$ and $u_{\mathrm{MS}}\left(x\right)$, we obtain the corresponded deformation maps $U_{\mathrm{SM}}\left(x\right)=x+u_{\mathrm{SM}}\left(x\right)$ and $U_{\mathrm{MS}}\left(x\right)=x+u_{\mathrm{MS}}\left(x\right)$. Unlike the regional deformation map, after the interpolation, these deformation maps are no longer diffeomorphic, and are not invertible. Next, we explain our method to overcome this shortcoming in our LG‐RBSN method.

### 2.4 Bijectivity constraints with residual compensation and demons registration

During spatial normalization the topology of a brain's anatomical structures should be preserved between healthy normal subjects, with connected neighboring morphological structures remain connected during deformation. Topology preservation is defined as a homeomorphic map that should be continuous, bijective, and inverse continuous. Topology preservation is a desired property for the estimated deformation field to perform a valid spatial normalization, because one‐to‐one and bijective correspondences of location structures between one brain and another are necessary requirements for generating biologically meaningful warped brains. A simple and fast solution to embed topology preservation into the deformation field is through considering simultaneously forward and reverse maps with certain symmetric constraints. Existing topology preserving methods use different strategies to achieve this goal. For instance, Thirion used an iteration scheme to compensate for half of the residual of the reverse warped forward transformation equally to both of the forward and reverse transformations until the residual reaches identity transformation (Thirion, 1998). Ashburner et al. (1999) used a Bayesian framework with a symmetric prior, so that the probability distribution of the forward and reverse deformations is identical. Inverse consistency constraint was proposed in (Christensen & Johnson, 2001), which used a symmetric cost function. Avants et al. proposed a symmetric diffeomorphic registration model where forward and reverse deformations meet at the middle of the registration (Avants et al., 2008), whereas Kuang used a cycle‐consistent design in a deep‐learning network to learn forward and reverse deformations concurrently (Kuang, 2019).

In our solution, the deformation field for each region is diffeomorphic with strict bijective constraints, but the combined deformation using IDW interpolation will no longer guarantee bijectivity. For example, moving local gyrus images needed to be cut to prevent overlap during warping (Pitiot et al., 2003). To address invertibility a poly‐affine method was proposed that composites local velocity vector fields associated with each local affine transformation, rather than compositing the displacement (Arsigny et al., 2005). However, this velocity vector field parametrization of the displacement is difficult for nonlinear deformation in our case. Alternatively, our solution uses a residual compensation method to impose bijective property into the existing nonbijective global warping fields $U_{\mathrm{SM}}\left(x\right)$ and $U_{\mathrm{MS}}\left(x\right)$, while using a demons registration to match sub‐cortical regions and the cerebral cortex mask. This prevents the mismatching of brain structures during residual compensation.

We use a residual compensation scheme to enforce bijectivity into both the direct forward deformation map $U_{\mathrm{SM}}\left(x\right)=x+u_{\mathrm{SM}}\left(x\right),x\in \Omega_{S}$ from S to M and the reverse deformation map $U_{\mathrm{MS}}\left(x\right)=x+u_{\mathrm{MS}}\left(x\right),x\in \Omega_{M}$ from M to S. At the same time, we use a demons registration method to match the brain's sub‐cortical regions (${\text{SubR}}^{i},i=1,\ldots ,n$) and cerebral cortex mask (CC). We have initial direct deformation map $U_{\mathrm{SM}}^{\left(0\right)}\left(x\right)=x+u_{\mathrm{SM}}^{\left(0\right)}\left(x\right)$ and initial reverse deformation map $U_{\mathrm{MS}}^{\left(0\right)}\left(x\right)=x+u_{\mathrm{MS}}^{\left(0\right)}\left(x\right)$. At each iteration t, we update both $u_{\mathrm{SM}}^{\left(t\right)}\left(x\right)$ and $u_{\mathrm{MS}}^{\left(t\right)}\left(x\right)$ according to following steps:

- Compute residual displacement
(4) $$r_{\mathrm{SM}}=u_{\mathrm{SM}}^{\left(t\right)}+u_{\mathrm{MS}}^{\left(t\right)}\circ U_{\mathrm{SM}}^{\left(t\right)}$$
(5) $$r_{\mathrm{MS}}=u_{\mathrm{MS}}^{\left(t\right)}+u_{\mathrm{SM}}^{\left(t\right)}\circ U_{\mathrm{MS}}^{\left(t\right)}$$
- Compute demons velocity for matching the brain's cerebral cortex mask
(6) $$v_{\mathrm{SM}}^{\mathrm{CC}}=\frac{\left(CC_{S}-CC_{M}\circ U_{\mathrm{SM}}^{\left(t\right)}\right)\nabla CC_{S}}{{\left\|\nabla CC_{S}\right\|}^{2}+\alpha {\left(CC_{S}-CC_{M}\circ U_{\mathrm{SM}}^{\left(t\right)}\right)}^{2}}$$
(7) $$v_{\mathrm{MS}}^{\mathrm{CC}}=\frac{\left(CC_{M}-CC_{S}\circ U_{\mathrm{MS}}^{\left(t\right)}\right)\nabla CC_{M}}{{\left\|\nabla CC_{M}\right\|}^{2}+\alpha {\left(CC_{M}-CC_{S}\circ U_{\mathrm{MS}}^{\left(t\right)}\right)}^{2}}$$
- Compute demons velocity for matching the brain's sub‐cortical regions
(8) $$v_{\mathrm{SM}}^{\text{SubR}}=\sum_{i=1}^{n}\frac{\left({\text{SubR}}_{S}^{i}-\mathrm{Sub}R_{M}^{i}\circ U_{\mathrm{SM}}^{\left(t\right)}\right)\nabla {\text{SubR}}_{S}^{i}}{{\left\|\nabla {\text{SubR}}_{S}^{i}\right\|}^{2}+\alpha {\left(\mathrm{Sub}R_{S}^{i}-\mathrm{Sub}R_{M}^{i}\circ U_{\mathrm{SM}}^{\left(t\right)}\right)}^{2}}$$
(9) $$v_{\mathrm{MS}}^{\text{SubR}}=\sum_{i=1}^{n}\frac{\left(\mathrm{Sub}R_{M}^{i}-\mathrm{Sub}R_{S}^{i}\circ U_{\mathrm{MS}}^{\left(t\right)}\right)\nabla \mathrm{Sub}R_{M}^{i}}{{\left\|\nabla \mathrm{Sub}R_{M}^{i}\right\|}^{2}+\alpha {\left(\mathrm{Sub}R_{M}^{i}-\mathrm{Sub}R_{S}^{i}\circ U_{\mathrm{MS}}^{\left(t\right)}\right)}^{2}}$$
- Update displacement fields

(10) $$u_{\mathrm{SM}}^{\left(t+1\right)}=u_{\mathrm{SM}}^{\left(t\right)}-w_{1}∙\beta ∙r_{\mathrm{SM}}-w_{2}∙G\left(\sigma^{2}\right)*v_{\mathrm{SM}}^{\mathrm{CC}}-w_{3}∙G\left({\sigma^{\prime }}^{2}\right)*v_{\mathrm{SM}}^{\text{SubR}}$$

(11) $$u_{\mathrm{MS}}^{\left(t+1\right)}=u_{\mathrm{MS}}^{\left(t\right)}-w_{1}∙\beta ∙r_{\mathrm{MS}}-w_{2}∙G\left(\sigma^{2}\right)*v_{\mathrm{MS}}^{\mathrm{CC}}-w_{3}∙G\left({\sigma^{\prime }}^{2}\right)*v_{\mathrm{MS}}^{\text{SubR}}$$

where, $w_{1}$, $w_{2}$, and $w_{3}$ are normalized weight with $w_{1}+w_{2}+w_{3}=1$, $G\left(\sigma^{2}\right)$ is a Gaussian kernel with variance $\sigma^{2}$. We will show in the experiment section that by using this approach we can significantly reduce the number of nonpositive Jacobian voxels after IDW interpolation.

### 2.5 Subjects and data acquisitions

All research procedures were performed in accordance with relevant guidelines and regulations as approved by the Columbia University Institutional Review Board. Forty two subjects (27/15 young/older, age [mean ± *SD*] = 25.11 ± 3.24/66.93 ± 3.71 years) were scanned using a Siemens Prisma 3‐Tesla MR scanner. T1‐weighted images were acquired using a magnetization‐prepared rapid gradient‐echo (MPRAGE) (TR = 2300 ms; TE = 2.32 ms; flip angle = 8°, voxel size = 1 mm × 1 mm × 1 mm; matrix size = 256 × 256, and 192 slices without gap). Task‐based functional MRI was acquired using a T2*‐weighted multiband gradient‐echo EPI (TR = 1 s; TE = 30 ms; flip angle = 62°, 64 slices without gap; slice thickness = 2 mm; 480 volumes; voxel size 2 mm × 2 mm × 2 mm, multiband factor = 4) pulse sequence. Another fMRI scan was acquired in the opposite phase encoding direction, which was used in this work solely for geometric distortion correction (GDC).

We employed an event‐related fMRI experimental task design. The task consisted of two ongoing stimuli: (1) A maximum contrast flashing checkerboard (i.e., visual stimulus) presented on either the right or left side of the screen, and (2) An alternating tone (i.e., auditory stimulus) paradigm played on either the right or left ear. The two sensory stimuli were presented with random onsets and durations (uniform distribution, range = 1.0–5.0 s). Overlaps between visual and audio stimuli were allowed, however, temporal overlapping of the bilateral presentation in the same modality was prohibited.

The data were collected in two runs; in the first run, subjects were instructed to attend to only one sensory stimulus (i.e., either visual or tonal) while ignoring the other. In the second run, they were instructed to attend the other sensory stimulus. Each scan consisted of 120 events: 60 events for visual and 60 for auditory stimulus. For each modality, 30 events on the right and 30 events on the left side spaced at inter‐stimulus‐intervals in the range of 1–17 s were drawn from a uniform distribution. Control for attention was achieved by asking the subjects to press a button twice with their right/left index finger (depending on the lateralization of the attended stimulus) as soon as the attended stimulus terminated. These responses were recorded during the entire scans. Throughout the experiment, subjects were required to maintain their gaze on a minuscule fixation spot in the center of the screen, and were given feedback on any incorrect or out‐of‐time responses by changing the color of the fixation spot from green to red. Eye fixation was monitored by recording the eye position and movement at all times using an eye‐tracking system. Subjects were trained multiple times outside of the scanner to learn and perform the task properly. All subjects learned the task correctly.

## 3 EXPERIMENTS AND RESULTS

In this section, we evaluate the performance of the proposed LG‐RBSN technique using simulated and real data. We first generated simulated 2D images of folded ribbons that resemble the folding patterns of the human cerebral cortex. Our main goal in this simulation was to show how the top performing volumetric normalization methods could fail in registering such simple 2D scenarios. We then extended our validation to experiments with real MRI and fMRI data targeting both primary visual and auditory regions. Our results were compared to a hybrid registration method (CVS) and a volumetric nonlinear whole brain registration method (ANTs) (http://www.picsl.upenn.edu/ANTS/, RRID:SCR_004757) that is considered the top performing nonlinear registration algorithm (Klein et al., 2009).

### 3.1 Simulation of cortical gyrus registration

**FIGURE 4:**
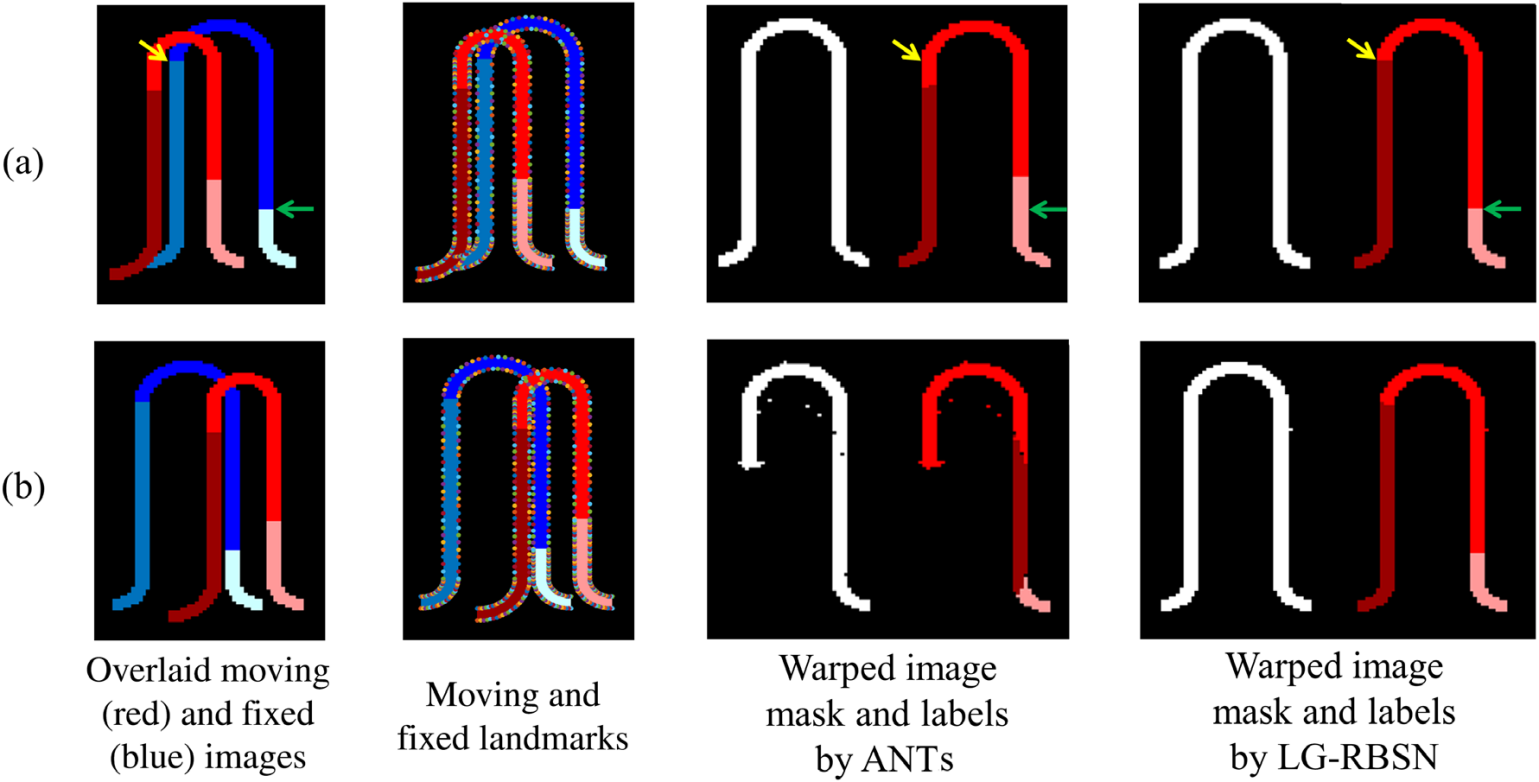
Comparison of ANTs and LG‐RBSN registration results in cortical gyrus registration simulations with cases of moving and fixed images (a) aligned initially and (b) mis‐aligned initially. In experiment (a), ANTs matched the whole gyrus mask perfectly but failed to accurately align the underlying three regions (yellow and green arrows mark the same location across images). In experiment (b), ANTs fell into a local minimum and failed to match even the binary mask of the entire gyrus. LG‐RBSN matched the whole structure mask and regions perfectly in both experiments. ANTs, advanced normalization tools; LG‐RBSN, landmark‐guided region‐based spatial normalization

To illustrate the problems associated with volumetric whole‐brain registrations and to demonstrate the effectiveness of our proposed solution to overcome those problems, we simulate a single cortical gyrus registration experiment in 2D. To best simulate the real task of cortical regions registration we assumed that both moving and fixed gyri have a similar shape and width (four pixels corresponding to 4 mm in most of the currently acquired T1‐weighted MRI scans), but are comprised of three regions of different lengths across the gyrus, as shown in the first column of Figure 4 where the three cortical regions are color‐coded for both images. The two rows in Figure 4 illustrate two simulated experiments with different initial positions: (a) with a relatively aligned initial position, and (b) with a mis‐aligned initial position. Please note that even with initial affine registration of the whole brain, many cortical features (sulci and gyri) could still remain completely misaligned. Therefore, the simulated initial positions of alignment and mis‐alignment between the moving and fixed images are very common in most spatial normalization or brain image registration scenarios. We used regional landmarks distributed at two sides of the ribbons in each region, as shown in the second column of Figure 4, to estimate a global bijective warping field using our method LG‐RBSN. We also applied a modified ANTs nonlinear registration pipeline from the half‐C to full‐C registration experiment to match the moving and fixed images (Avants et al., 2014).

In experiment (a), both LG‐RBSN and ANTs resulted in a comparable overlap between the entire gyrus structure (ANTs: 99.11% vs. LG‐RBSN: 99.86%), shown as a binary mask in the third and last columns of Figure 4. However, such high correspondence for the entire gyrus, did not hold for the three comprising regions and the average regional DSC dropped from 99.91% for LG‐RBSN to 86.39% for ANTs. This is because intensity‐based volumetric registrations, such as ANTs, cannot discriminate adjacent cortical regions. Therefore, the underlying three regions will not necessarily be registered, as shown in the third column of Figure 4. Alternatively, LG‐RBSN almost perfectly aligns the underlying regions, as shown in the last column of Figure 4.

In experiment (b), unlike LG‐RBSN which generated a perfect correspondence, ANTs failed to generate an acceptable overlap even on the binary mask of the entire gyrus (ANTs: 82.49% vs. LG‐RBSN: 99.66%), as seen in third and last columns of Figure 4. As mentioned in the introduction, the ANTs failure is due to its vulnerability to the local minimum during optimization. The global affine initial alignment failed to provide a sufficient initial alignment of cortical regions, which is often the case in any nonlinear registration problem. Consequently, the underlying three regions drastically failed to correspond when using ANTs methods (ANTs: 42.60% vs. LG‐RBSN: 99.66%), as seen in the third and last columns of Figure 4. These results highlight the importance of detecting a true optimum point in any nonlinear registration method.

Jacobian matrix is commonly used to evaluate the diffeomorphic property of the warping field, as the local deformation is invertible and preserves the topology only at locations with positive Jacobian determinant. Table 1 also lists the number of voxels with nonpositive Jacobians in each registration method. While we enforce all regional warping fields to be topology preserving by imposing bijectivity during the regional registration process, the combined global warping field is not guaranteed to be topology preserving due to the sharp transitions between some neighboring regions. To address this issue, we used residual compensation method to impose bijectivity to the obtained global warping field. To evaluate the performance of the utilized method, we used the number of voxels with nonpositive Jacobian. As it seen in the Table 1, ANTs produces 217 pixels with nonpositive Jacobian determinant in experiment (b), whereas all pixels show positive Jacobian determinant when LG‐RBSN is being used for registration, emphasizing the performance of a residual compensation method in our LG‐RBSN using simulated data.

**TABLE 1:** DSC between warped and fixed images of using ANTs and LG‐RBSN in the simulation cases of (a) aligned and (b) mis‐aligned initially

Experiments	DSC between warped moving and fixed binary mask of all regions		Average DSC between warped moving and fixed image labels		Number of nonpositive Jacobian pixels	
	ANTs	LG‐RBSN	ANTs	LG‐RBSN	ANTs	LG‐RBSN
(a)	99.11%	99.86%	86.39%	99.91%	0	0
(b)	82.49%	99.66%	42.60%	99.66%	217	0

Abbreviations: ANTs, advanced normalization tools; DSC, dice similarity coefficient; LG‐RBSN, landmark‐guided region‐based spatial normalization.

### 3.2 Evaluation using human brain structural images

**FIGURE 5:**
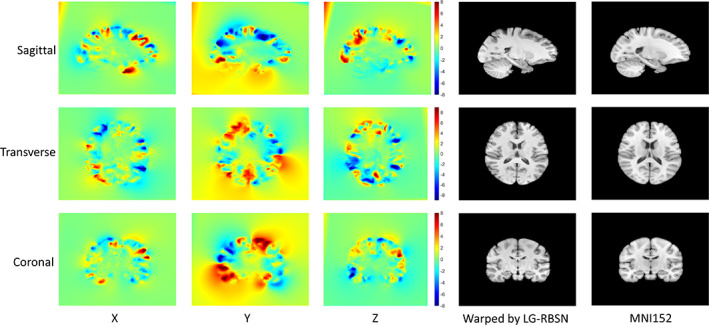
Visualization of LG‐RBSN estimated global displacement vector field of one example subject. The X, Y, and Z denote the displacement in each direction with a unit of millimeter. This displacement vector field has a maximum displacement of 16.1 and a minimum displacement of −17.3, and is mostly within a range between −8 and 8. The visualization shows that our method is capable to work with large and localized displacements. LG‐RBSN: landmark‐guided region‐based spatial normalization

**FIGURE 6:**
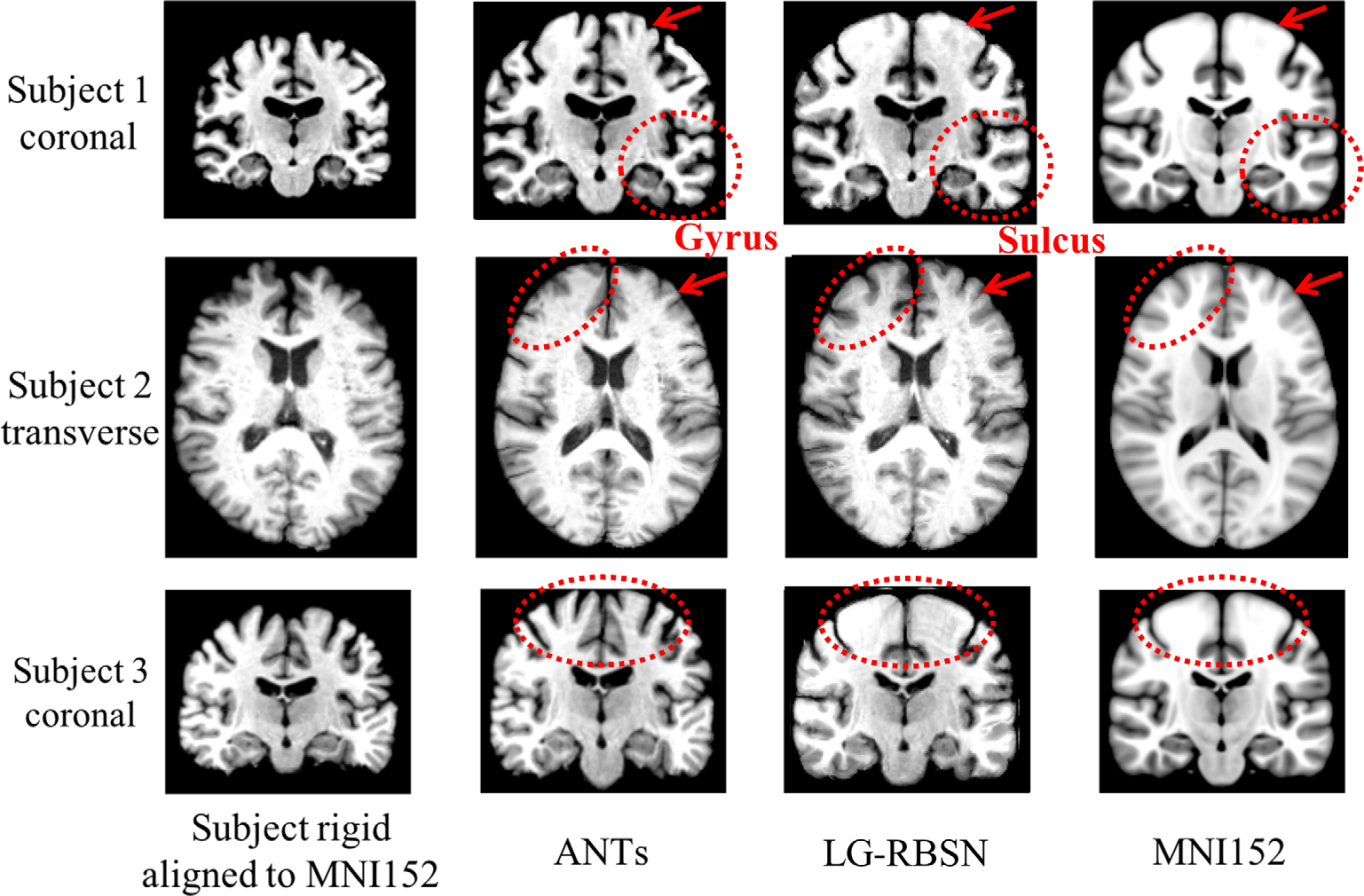
Spatial normalized brain qualification evaluation comparison between LG‐RBSN and ANTs. The first and the last columns illustrate subjects' brain images rigid aligned (showing as moving images) to MNI152 brain images (as fixed images). The second and the third columns illustrate subjects' brain images after ANTs/LG‐RBSN nonlinear registration to MNI152 brain images. LG‐RBSN shows clearly better performance compared to ANTs in red dotted circles highlighted areas. In subject 2, ANTs mismatched a gyrus of the subject's cerebral cortex to a sulcus in MNI152 space, whereas LG‐RBSN matched the corresponding sulci properly. ANTs, advanced normalization tools; LG‐RBSN, landmark‐guided region‐based spatial normalization

Using our LG‐RBSN solution we estimated a global warping field between each subject, described in Section 2.5, and the MNI152 template utilizing regional WM and pial surfaces vertices to extract corresponding landmarks. Figure 5 shows the estimated global warping field between one typical subject and the MNI152 using LG‐RBSN solution. For comparison, we also used ANTs (deformation model: SyN; similarity: normalized mutual information; regularization: Gaussian smoothing) to perform the same registration. For qualitative evaluation of our method and ANTs, the two obtained global warping fields were applied to each subject's T1‐weighted structural brain image, with results shown in Figure 6 for three selected subjects. LG‐RBSN shows a clear improvement in aligning brain cortical regions as highlighted by red dotted circles in Figure 6. The arrows in Figure 6 show how a sulcus can be generated by ANTs where the target image does not have such a structure (Subject 1), and how ANTs mismatched a gyrus of the subject to a sulcus in MNI152. Our method on the other hand properly matched the corresponding sulcus of the subject to the sulcus in MNI152 (Subject 2). This is because of performing optimization in 3D Euclidean space to find correspondence of the brain, which is often used in volumetric registration methods including ANTs. A small shift in the volume space can mismatch two functionally distinct locations of the brain, whereas our solution uses spherical registration to find the correspondence that directly matches brain folding patterns in each brain region, independently.

**FIGURE 7:**
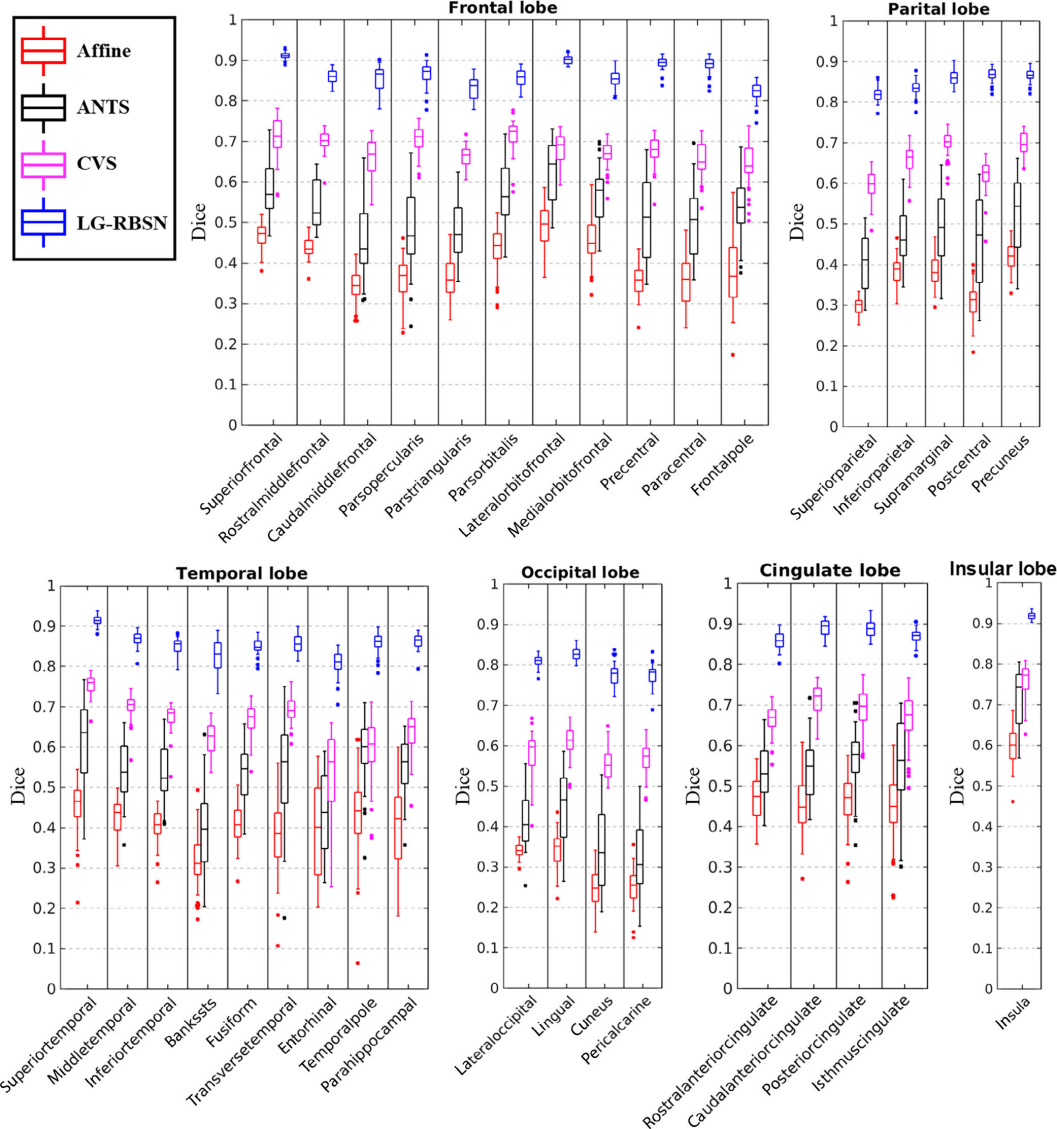
Cortical regional DSC comparison between affine, ANTs, CVS, and LG‐RBSN in different brain lobes. LG‐RBSN shows significantly higher DSC in matching brain cortical regions than ANTs and CVS. ANTs, advanced normalization tools; CVS, combined volumetric and surface registration; DSC: Dice similarity coefficient; LG‐RBSN, landmark‐guided region‐based spatial normalization

**FIGURE 8:**
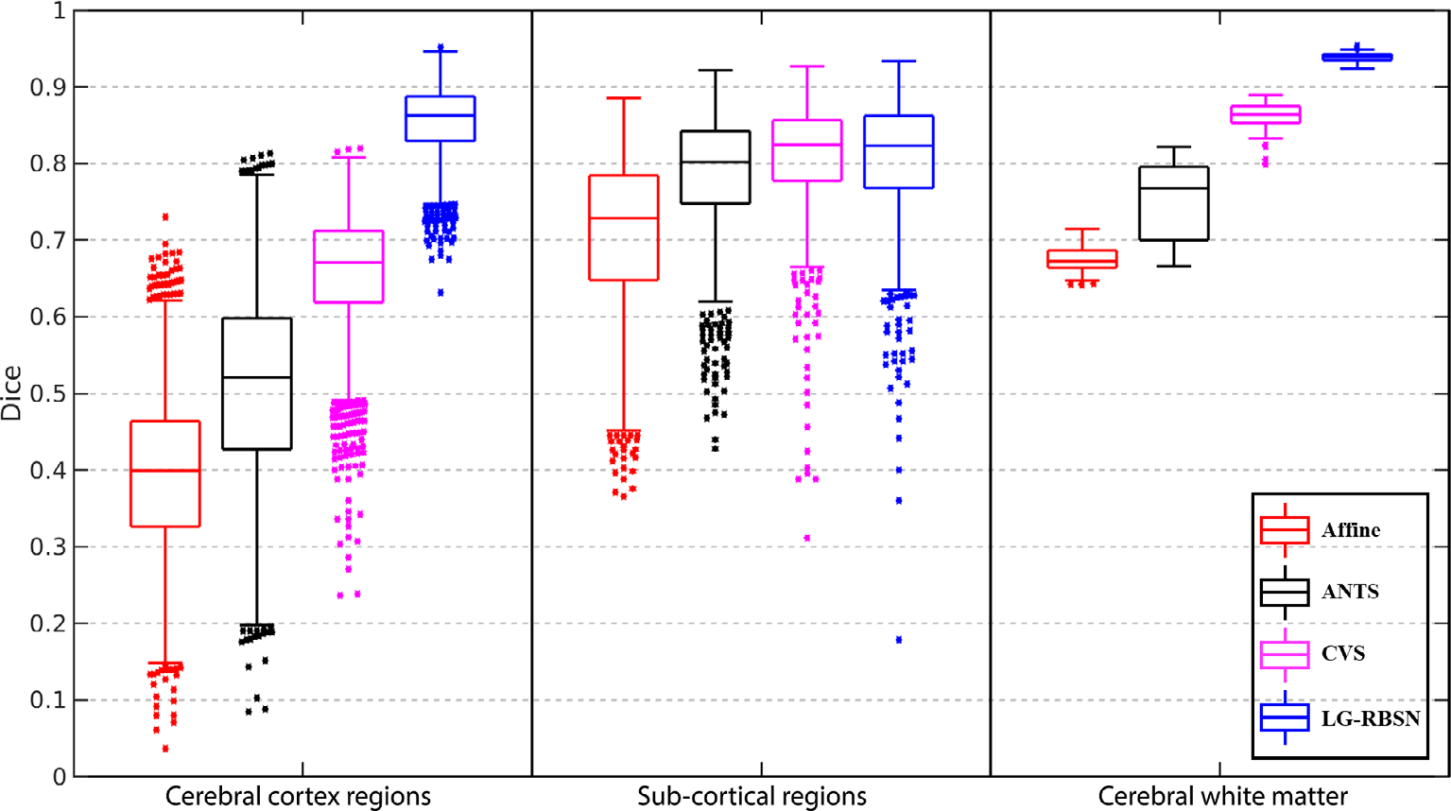
DSC comparison between affine, ANTs, CVS, and LG‐RBSN. LG‐RBSN shows significantly higher DSC in matching brain cortical regions, sub‐cortical regions, and cerebral WM than ANTs. LG‐RBSN shows significantly higher DSC in matching brain cortical regions and cerebral WM than CVS. And LG‐RBSN is more robust working with both young and older subjects compared to ANTs and CVS, as LG‐RBSN shows less variance of DSC in matching brain cortical regions and cerebral WM. ANTs: advanced normalization tools; CVS: combined volumetric and surface registration; DSC: dice similarity coefficient; LG‐RBSN: landmark‐guided region‐based spatial normalization; WM, white matter

For quantitative evaluation, we first evaluate the first two blocks of the algorithm (Sections 2.1 and 2.2 in Figure 1), which generate the regional warping fields after the landmark matching and regional large deformation diffeomorphic registration. These regional warping fields can accurately match brain cortical regions with an averaged DSC of 0.8 (He & Razlighi, 2020), which validated the accuracy and efficiency of the initial FreeSurfer surface registration, landmark down‐sampling, automatic landmark‐matching and the regional diffeomorphic registration steps. Although, these regional warping fields can match cortical regions accurately, but are limited to be only applicable to each region independently instead of the whole brain. As independently warping each region can cause overlaps and gaps between regions after the deformation, regional warping fields are not ideal for the application of brain structures and the functional brain activation patterns, which typically cover multiple brain regions. Thus, we introduced the IDW interpolation and bijectivity constraints steps to overcome these problems. Finally, for the quantitative evaluation of the global warping fields after all the steps of the algorithm, we used the global warping fields obtained above to warp each subject's FreeSurfer delineated regions, described in Section 2, onto MNI152 space (cortical regions include: banks of superior temporal sulcus, caudal anterior cingulate, caudal middle frontal, corpus callosum, cuneus, entorhinal, fusiform, inferior parietal, inferior temporal, isthmus cingulate, lateral occipital, lateral orbitofrontal, lingual, medial orbitofrontal, middle temporal, parahippocampal, paracentral, pars opercularis, pars orbitalis, pars triangularis, pericalcarine, postcentral, posterior cingulate, precentral, precuneus, rostral anterior cingulate, rostral middle frontal, superior frontal, superior parietal, superior temporal, supramarginal, frontal pole, temporal pole, transverse temporal, and insula). As done previously (Balakrishnan et al., 2019; Klein et al., 2009; Yang et al., 2017), we have evaluated our registration accuracy using the DSC between the regional binary masks of the warped and corresponding target regions. We chose FreeSurfer because it has been shown to produce reliable parcellations of the cortex with a high accuracy compared to manual delineation of cortical regions (Desikan et al., 2006); However, any other consistent parcellation technique can be used for this underlying segmentation of the human brain. For comparison, we also applied affine registration (similarity as correlation ratio), ANTs (deformation model: SyN; similarity: normalized mutual information; regularization: Gaussian smoothing), and CVS (default settings). Figure 7 illustrates the distribution of the DSC between corresponding regions for all cortical regions in FreeSurfer using boxplot for Affine (in red), ANTs (in black), CVS (in magenta), and LG‐RBSN (in blue) categorized in six lobar brain segments. As shown in Figure 7, LG‐RBSN significantly outperforms ANTs and CVS in all cortical regions reaching to highest DSC in the insula region with (DSC = 0.9185 ± 0.0116 (mean ± SD); ANTs: improved by 28.35% (*p* = 1.59e−31); CVS: improved by 20.84% (*p* = 6.24e−39)) and the lowest DSC in the pericalcarine region (DSC = 0.7737 ± 0.0379 (mean ± SD); ANTs: improved by 140.88% (*p* = 1.76e−50); CVS: improved by 37.15% (*p* = 1.77e−43)). Compared to ANTs, as for the improvement in lobar segments of the brain, our results show that the highest improvement was achieved in the occipital lobe (DSC improved by 108.36%; *p* = 1.91e−52) with the least improvement in the insular lobe (DSC improved by 28.35%; *p* = 1.59e−31). Compared to CVS, the highest improvement was also achieved in the occipital lobe (DSC improved by 38.06%; *p* = 3.42e–53) with the least improvement also in the insular lobe (DSC improved by 20.84%; *p* = 6.24–39). As shown in Figure 8, in total, using LG‐RBSN has substantially improved the correspondence between cortical regions (DSC = 0.8558 ± 0.0080 (mean ± SD)), which is significantly higher (DSC improved by 67.30%; *p* = 1.23e−50) than the results obtained by ANTs (DSC = 0.5115 ± 0.0641; mean ± SD), and is also significantly higher (DSC improved by 29.80%; *p* = 1.95e−69) than the results obtained by CVS (DSC = 0.6593 ± 0.0197; mean ± SD). Comparing the improvement of cortical regions correspondence in young and older subjects, LG‐RBSN outperforms ANTs in both groups with a significantly higher improvement (*p* = .0042) in older subjects (DSC improved by 75.04%) than the improvement in young subjects (DSC improved by 63.25%).

Next, we evaluated the effectiveness of the LG‐RBSN for aligning the sub‐cortical regions and cerebral WM in comparison with the results obtained by using ANTs and CVS (segmented with FreeSurfer, sub‐cortical regions include lateral ventricle, ventral DC, cerebellum white matter, cerebellum cortex, thalamus, caudate, putamen, pallidum, hippocampus, amygdala, third ventricle, and brain stem). Figure 8 shows the results of this evaluation along with the evaluation of all cortical regions obtained above. As shown in Figure 8, our method significantly (*p* = 8.76e−03) outperformed ANTs in matching sub‐cortical regions with a higher DSC value (0.8056 ± 0.0267; mean ± SD) compared to DSC values when using ANTs (0.7840 ± 0.0446; mean ± SD). And our method shows comparable performance with CVS in matching sub‐cortical regions (*p* = .41). Furthermore, our method shows significantly improved DSC (0.9387 ± 0.0046; mean ± SD) of matching cerebral WM mask (ANTs: DSC improved by 25.56%, *p* = 9.33e−40; CVS: DSC improved by 9.00%, *p* = 5.48–53), compared to the DSC of using ANTs (0.7477 ± 0.0499; mean ± SD) and compared to the DSC of using CVS (0.8613 ± 0.0186; mean ± SD).

**FIGURE 9:**
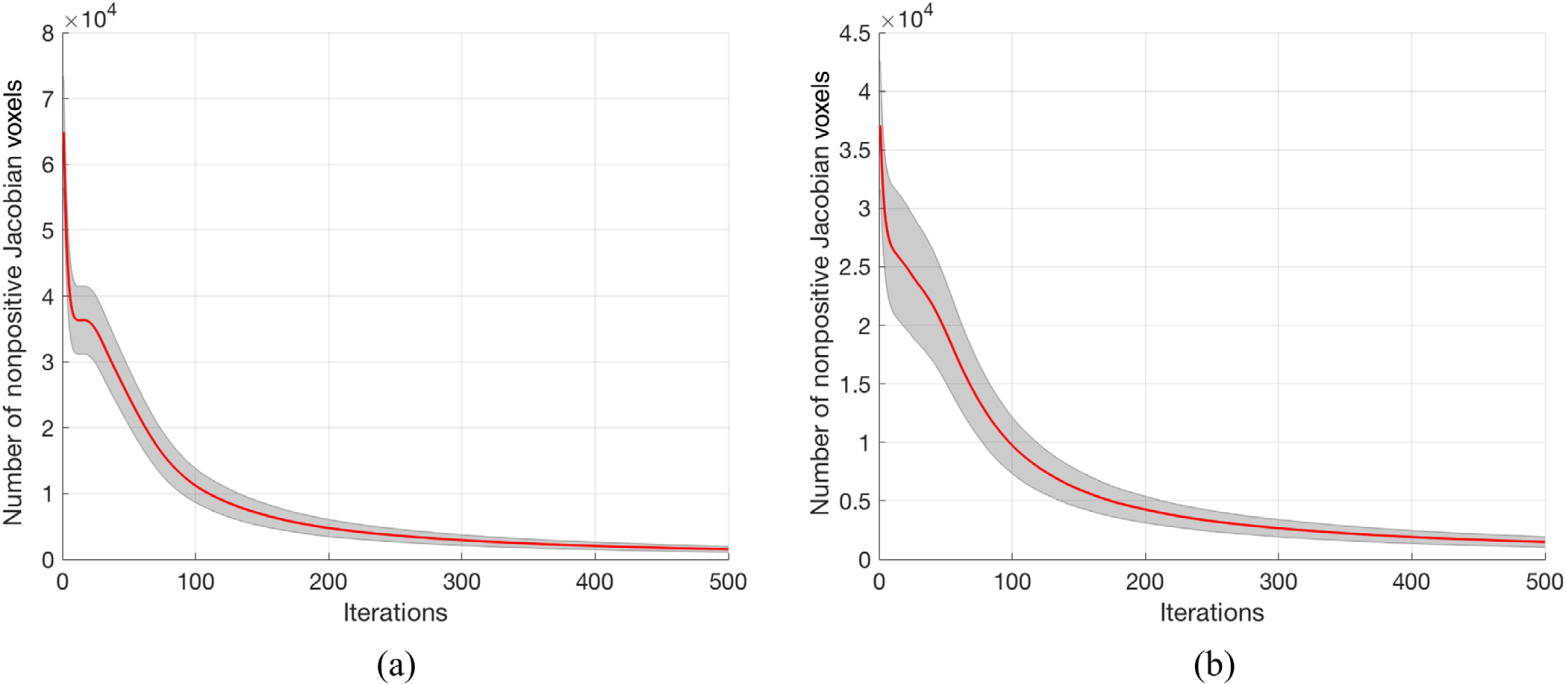
Number of nonpositive Jacobian voxels decreases along bijectivity constrain iterations for (a) forward (subject to MNI152) (b) backward (MNI152 to subject) warping field. The red curve and the grey region represent the mean and the standard deviation

Akin to the simulation section, the number of nonpositive Jacobian voxels is used to quantify the bijectivity property of the global warping field, with a smaller number of nonpositive Jacobian voxels indicates better bijectivity property. To evaluate the last block of the algorithm (Section 2.4 in Figure 1), which is the bijectivity constrain step, we calculated the number of nonpositive Jacobian voxels along with iterations. Results are shown in Figure 9. The final global warping fields estimated by using our solution have 1338.1 ± 404.3 (mean ± SD) nonpositive Jacobian voxels for the forward warping field and 1318.3 ± 405.9 (mean ± SD) voxels for the backward warping field, compared to ANTs with 1483.2 ± 2121.1 (mean ± SD) for the forward warping field and 1473.9 ± 2239.1 (mean ± SD) for the backward warping field. There is no significant difference between the number of nonpositive Jacobian voxels between ANTs and LG‐RBSN for both forward (*t* = 0.4354; *p* = .6644) and backward (*t* = 0.4433; *p* = .6587) warping fields. Compared with LG‐RBSN and ANTs, CVS has a greater number of nonpositive Jacobian voxels (7204.0 ± 5591.2; mean ± SD) with worse bijectivity. However, for LG‐RBSN, the number of nonpositive Jacobian voxels can be lowered even to reach zero by using our residual compensation method, with a small cost in the accuracy. We will discuss the tradeoff between accuracy and the regularization in Section 4.

### 3.3 Evaluation using human brain functional images

We have shown in the previous section that LG‐RBSN significantly improves the regional correspondence between warped and reference images. However, that does not necessarily imply that the enhancement will be directly transformed to functional imaging data. As we have shown previously in a preliminary study (Razlighi, 2016), the improvement in the structural overlap had significantly increased the statistics of the group‐level brain activations in primary visual cortex, but it did not generalize to the group‐level activations from the primary auditory cortex. We again applied LG‐RBSN to the statistical parametric maps obtained from both an auditory and a visual fMRI experiment to generate group‐level activation maps.

**FIGURE 10:**
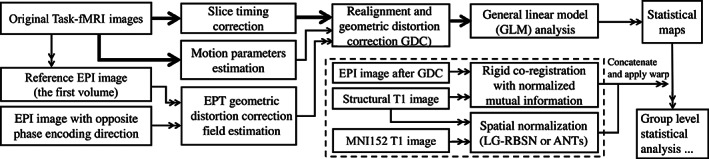
The processing pipeline for task‐based fMRI data. The thick arrows show the transfer of 4D fMRI data, the double thin arrow shows the transfer of the 3D data, and the thin arrow shows the transfer of the parameters. ANTs, advanced normalization tools; EPI, echo planar imaging; LG‐RBSN, landmark‐guided region‐based spatial normalization

We have compared the obtained activation maps to those generated using Affine, ANTs, and CVS, and to the anatomy of the primary visual and auditory cortices, where we expect to detect the true‐positive activations. The preprocessing pipeline for the task‐based fMRI data is illustrated in Figure 10. Briefly, slice timing correction is applied to the raw fMRI timeseries to account for the difference in the acquisition delay between slices (Parker et al., 2017; Parker & Razlighi, 2019). At the same time, motion parameters are estimated on raw fMRI scans using rigid‐body registrations performed on all the volumes in reference to the first volume. Additionally, the first volumes are extracted from another fMRI scans with opposite phase encoding directions to estimate the GDC field using a susceptibility‐induced distortions correction technique called topup (Andersson et al., 2003) provided in FSL software package (https://fsl.fmrib.ox.ac.uk/fsl, RRID:SCR_002823; Smith et al., 2004). Then, the estimated motion parameters and geometric distortion field are combined and applied to the slice timing corrected fMRI time‐series to get the distortion and motion‐corrected fMRI time‐series. First level general linear modeling is performed independently on each voxel using multiple regression with four variables of interest (stimuli timing convolved with canonical Double‐Gamma hemodynamic response function) resulting to four different statistical parametric maps which will be warped onto a standard space to be able to perform group‐level statistical analysis. Each subject's global registration warping field estimated in the previous experiment was concatenated with the within‐subject functional to structural rigid‐body transformation, and used to project brain auditory/visual activation statistical maps of that individual into MNI152 space for group‐level analysis. Group‐level analysis was done by simple regression where a voxel was deemed active if its averaged point‐estimates were significantly different from zero.

**FIGURE 11:**
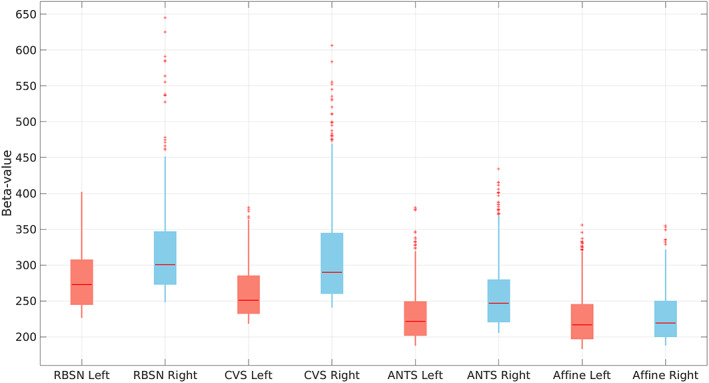
Distribution of the point estimates for visual task‐based fMRI group level visual activation in brain contralateral occipital lobe using different spatial normalization methods (left: for stimulating left visual hemifield; right: For stimulating right visual hemifield). ANTs, advanced normalization tools; CVS, combined volumetric and surface registration; LG‐RBSN, landmark‐guided region‐based spatial normalization

We first compared the actual statistics of the group‐level analysis results when LG‐RBSN was used as the spatial normalization versus the results obtained with using Affine, ANTs, and CVS. The statistics are quantified as the point estimates (*β* values) of the 1000 activated voxels with the highest *z*‐statistics in the brain's left lateral occipital cortex for stimulating right visual hemifield and vice versa. Figure 11 illustrates the distribution of the point‐estimates in the top 1000 activated voxels using boxplots (left: for stimulating left visual hemifield; right: for stimulating right visual hemifield). As seen in Figure 11, the mean point estimates of group‐level brain activation using our method (left: *β* = 280.20 ± 40.96, right: *β* = 315.33 ± 57.34) is significantly higher (left: *t* = 30.08 *p* = 0, right: *t* = 26.32 *p* = 0) than that obtained by ANTs (left: *β* = 229.24 ± 34.54, right: *β* = 255.76 ± 42.84), and is also significantly higher than that obtained by CVS for the left visual hemifield stimuli (*β* = 261.90 ± 35.70; *p* = 8.62e−26) but is not significantly higher for the right hemifield stimuli (*β* = 309.98 ± 64.71; *p* = 5.06e−2).

**FIGURE 12:**
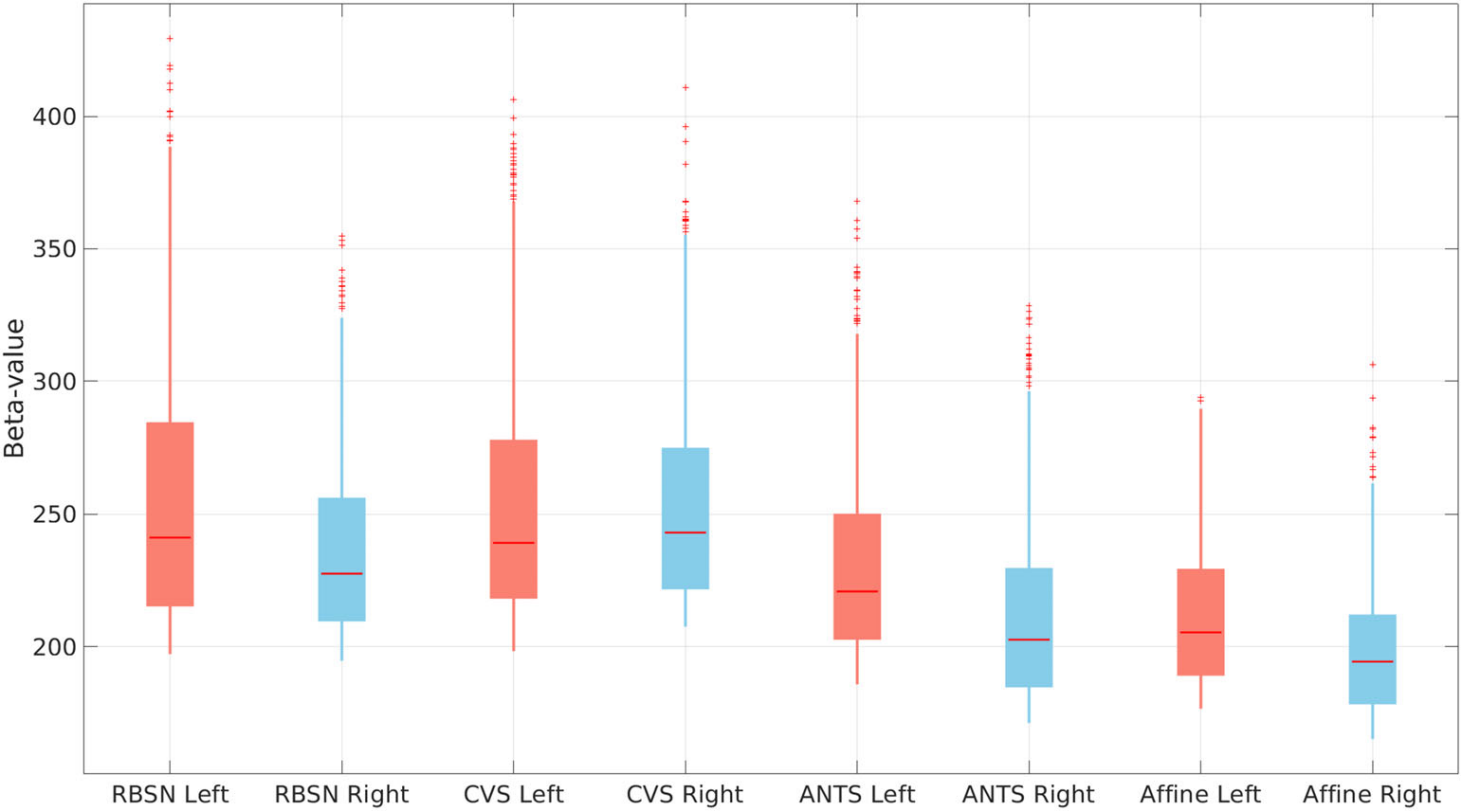
Beta values of tonal task fMRI group level auditory activation in contralateral transverse temporal cortex and superior temporal cortex using different spatial normalization methods (left: for stimulating left ear; right: for stimulating right ear). ANTs, advanced normalization tools; CVS, combined volumetric and surface registration; LG‐RBSN, landmark‐guided region‐based spatial normalization

For auditory stimulation we used combination of two FreeSurfer regions (transverse temporal gyrus and superior temporal gyrus) to generate a binary mask for the primary auditory cortex. Figure 12 illustrates the distribution of the point estimates from the 1000 voxels with highest significance level within the binary mask using boxplots and also shows the comparison between the point estimates obtained by our LG‐RBSN solution and the ones obtained by using Affine, ANTs, and CVS (left: for stimulating left ear; right: for stimulating right ear). As seen in this figure, the mean point estimates of the group level brain activation using our method (left: *β* = 255.22 ± 48.84, right: *β* = 236.28 ± 33.02) is significantly higher (left: *t* = 12.89, *p* = 1.40e−36; right *t* = 16.68, *p* = 0) than that obtained by ANTs (left: *β* = 230.46 ± 36.12, right: *β* = 211.48 ± 33.47). Compared to CVS, the mean point estimates using LG‐RBSN is slightly higher than that obtained by CVS for the left ear stimuli (*β* = 253.56 ± 45.97; *p* = .43) but is significantly lower for the right ear stimuli (*β* = 253.58 ± 39.08; *p* = 5.79e−26). As compared to ANTs, the group level statistics results in both visual and auditory task‐based fMRI indicate that the magnitude of the fMRI signal will significantly increase if we use a more accurate spatial normalization technique (either CVS or LG‐RBSN). When comparing CVS and LG‐RBSN, results indicate that the improvement in the regional correspondence would improve the group‐level activation statistics in visual and left ear auditory stimuli evoked activation, but the regional correspondence improvement did not transform to the increase in the group‐level activation statistics of right ear auditory stimuli evoked activation. This might be due to the misalignment between the brain morphology and the underlying functional architecture, which will be discussed later in Section 4.

Improving the group‐level statistics in our fMRI experiments shows that the proposed spatial normalization technique increases the statistical power to detect smaller effects that may not be detectable with the conventional methods such as ANTs. Still, the method does not guarantee that the false‐positive rate will not increase as well, which is often the case in many advanced developments for fMRI processing. To address this problem, we use receiver operating characteristic (ROC) curves which associate the cost of improvement in the true‐positive rate (sensitivity) to the false‐positive rate (1 − specificity) at different threshold levels for detecting an effect. However, using ROC curve for evaluating any fMRI experiment is a challenging task due to the lack of gold standard measurement. In this article, we use the masks of the primary visual cortex (lateral occipital) and auditory cortex (transverse temporal gyrus and superior temporal gyrus), given by FreeSurfer, as the regions that we expect to see activated voxels in and any detection of activated voxels outside these masks can be considered as false‐positive. Therefore, true‐positive rate is the number of activated voxels divided by the total number of voxels inside the region, and false‐positive rate is the number of activated voxels in the vicinity of the regional masks (obtained by dilating the same regional masks) divided by the total number of voxels in the dilated regions. By changing the threshold for significance (*t*‐statistics ranging from 0 to 15), we plot the curve illustrating the association between these two rates. The area under the curve (AUC) of the ROC is often used as the main performance metric for quantitative comparison.

**FIGURE 13:**
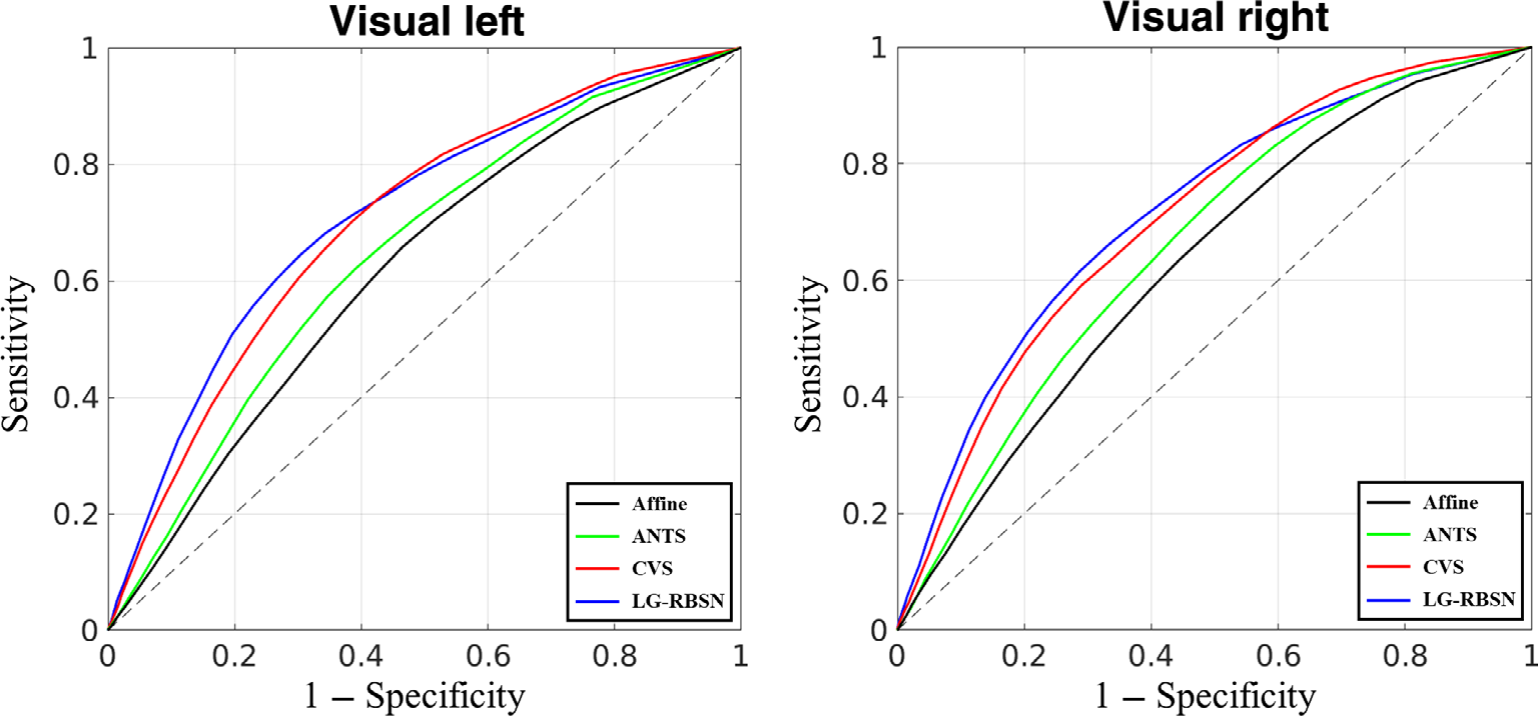
ROC curve evaluating spatial normalization methods with visual task‐based fMRI group level *t*‐statistics activation map compared to FreeSurfer lateral‐occipital region (left: for stimulating left visual hemifield; right: for stimulating right visual hemifield). ANTs, advanced normalization tools; CVS, combined volumetric and surface registration; LG‐RBSN, landmark‐guided region‐based spatial normalization; ROC, receiver operating characteristic

Figure 13 illustrates the ROC curves obtained for the two visual stimuli (left plot: for stimulating left visual hemifield; right plot: for stimulating right visual hemifield) when LG‐RBSN were used (blue curve) versus Affine (black curve), ANTs (green curve), and CVS (red curve). Our LG‐RBSN method shows an AUC equal to 0.7124/0.7180 for left/right hemifield visual stimulation which demonstrates about 10.24%/8.23% improvement in comparison to the AUC obtained from ANTs ROC (0.6462/0.6634 for left/right visual hemifield stimulation). And LG‐RBSN also outperforms CVS (0.7022/0.7086 for left/right hemifield visual stimulation). This result indicates that such improvement in the sensitivity of our proposed method is not at the expense of an increased false‐positive rate.

**FIGURE 14:**
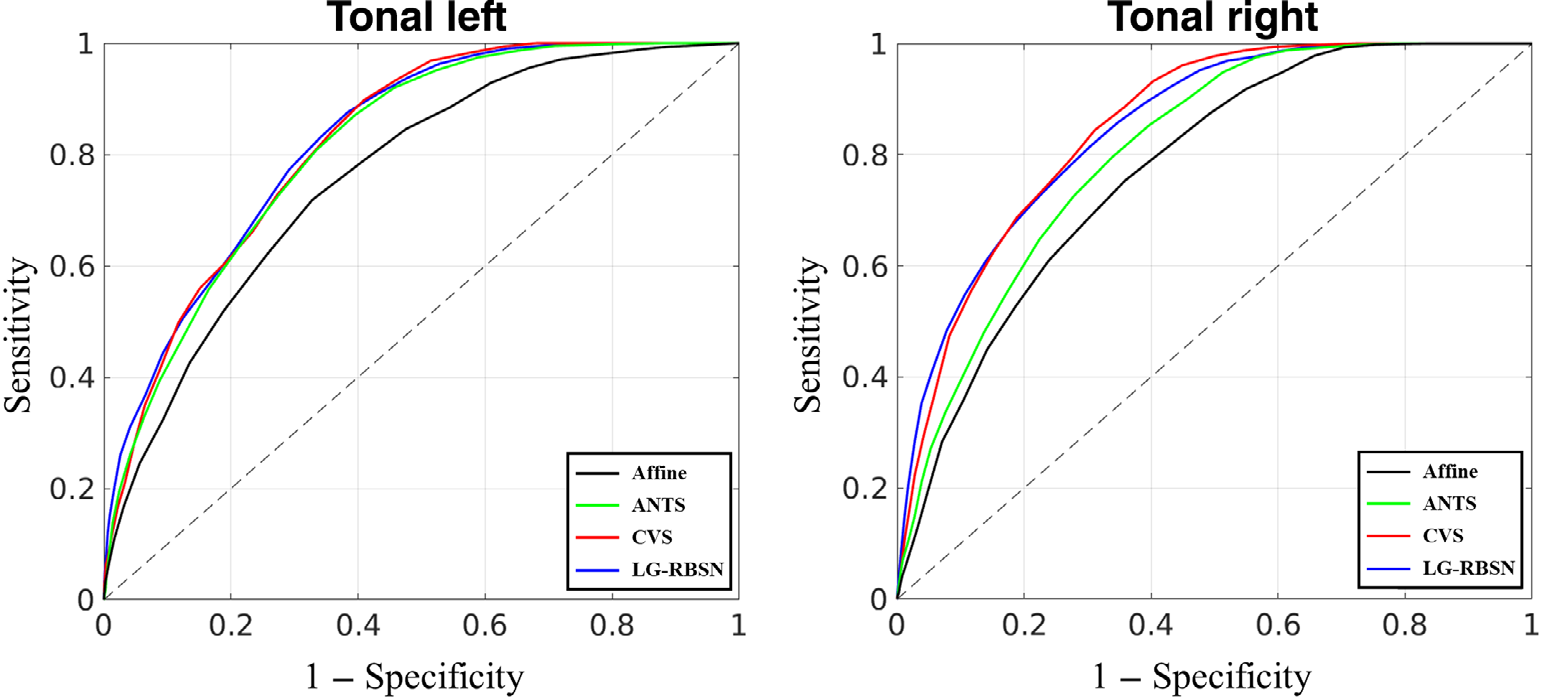
ROC curve evaluating spatial normalization methods with auditory task‐fMRI group level *t*‐statistics activation map compared to FreeSurfer segmented neuroanatomical brain contralateral primary auditory mask (left: for stimulating left ear; right: for stimulating right ear). ANTs, advanced normalization tools; CVS, combined volumetric and surface registration; LG‐RBSN, landmark‐guided region‐based spatial normalization; ROC, receiver operating characteristic

Figure 14 illustrates the ROC curves obtained for the two auditory stimuli (left plot: for stimulating left ear; right plot: for stimulating right ear) when LG‐RBSN was used (blue curve) versus Affine (black curve), ANTs (green curve), and CVS (red curve). LG‐RBSN shows an AUC equal to 0.8276/0.8490 for left/right ear auditory stimulation which demonstrates about 1.47%/5.10% improvement in comparison to the AUC obtained from ANTs ROC (0.8156/0.8078 for left/right ear auditory stimulation). As compared to CVS (0.8238/0.8513 for left/right ear auditory stimulation), LG‐RBSN shows a slightly higher (0.46%) AUC for the left ear auditory stimulation, but a slightly lower (−0.27%) AUC for the right ear auditory stimulation. As compared to ANTs, the results again indicate that such improvement in the sensitivity of our proposed method is not accompanied by an increase in the false‐positive rate. But similar to the previous results on the point estimates, when comparing LG‐RBSN and CVS, regional correspondence improvement did not transform to the increase in the sensitivity and specificity for the group‐level activation statistics for the right ear auditory stimuli. As discussed previously, one reason might be the misalignment between the brain morphology and the underlying functional architecture. Due to the lack of gold standard, another explanation might be the assumption we made when defining the primary auditory cortex, where we treated as the expected location of true‐positive activations.

Taken together, results in Figures 13 and 14 highlight the superiority of the proposed LG‐RBSN solution in detecting small effects that were not detectable using conventional methods such as ANTs, without increasing the false‐positive rate, which is the ultimate goal in any advancement in method development for fMRI processing pipeline. Further functional evaluation with fMRI data between LG‐RBSN and CVS might be needed with more detailed brain parcellations such as HCP MMP (Glasser et al., 2016), or using other task stimuli with more specific activation patterns such as face stimuli and the fusiform face area.

### 3.4 Coding and execution time

Most of the implementation in this work is done with Matlab R2017b. We have shared the code for LG‐RBSN solution on our laboratory website (https://qnlab.weill.cornell.edu/research/preprocessing-fmri-data) as well as our laboratory *github* repository page (https://github.com/QuantitativeNeuroimagingLaboratory). The current implementation of the LG‐RBSN takes an average of around 18 h to be performed on each region. However, since the registration of each region is done independently, they all can be done in parallel using cluster of high‐performance computing with around 100 number of cores. Using the cluster makes the total execution time to be around 75 h for each subject's brain registration. However, applying the obtained warping field takes no more time than applying other conventional spatial normalization methods.

## 4 DISCUSSION

We presented a novel region‐based volumetric spatial normalization solution for human structural and functional brain image processing and statistical analysis. Compared to typical volume‐based spatial normalization methods that use intensity‐based similarity measurements, our solution uses landmark guidance which specifies a concrete spatial correspondence in the volumetric space based on matching of the brain cortical folding patterns. To address the local minimum problem in optimization steps in most of the whole brain volume‐ or surface‐based, or hybrid image registrations, we propose to independently estimate a diffeomorphic warping field for each cortical region. Locally‐affine and poly‐affine registration methods have been previously used in medical image registration (Arsigny et al., 2005; Porras et al., 2018). However, here we propose a novel region‐based nonlinear registration approach and present the first study in combing the regional nonlinear warping fields. To generate a single smooth global warping field with a smooth transition across adjacent regions, we propose to use IDW interpolation. Compared to other symmetric registration algorithms (Ashburner et al., 1999; Avants et al., 2008; Christensen & Johnson, 2001), which regularize the bijectivity property during registration, our proposed residual compensation method is applicable to any given nontopology‐preserving warping fields, as long as both forward and backward warpings are available.

Compared to typical surface‐based spatial normalization methods that only warp brain cortical surface, our solution extended surface‐based methods to estimate a volumetric warping field. This method is considered more robust than typical surface‐based spatial normalization methods, since fMRI brain activations are captured originally in the 3D Euclidean space and thus avoiding the projection of the volumetric data onto the cortical surface. Furthermore, surface‐based methods are more susceptible to the inaccuracies that often occur during reconstructions of cortical surface due to geometric distortion, especially in high‐field and multiband acquisitions. While any inaccuracy in the surface reconstruction will cause surface‐based method to include regions from outside of the brain or the white matter, our solution computes volumetric deformation mappings that are applied to all gray‐matter, white‐matter, and other brain regions, thus preventing loss of fMRI data due to inaccurate surface extraction. In future work, we will aim at a quantitative evaluation between our volume‐based registration method and other surface‐based registration methods (e.g., FreeSurfer), however a direct comparison is challenging as the group activation results are in different space (Tucholka et al., 2012). The comparison between surface‐based and volume‐based spatial normalization is beyond the scope of this article.

Evaluating spatial normalization methods has received considerable attention in recent years, which are typically evaluated by measuring the overlap between the warped anatomical regions and the counterpart regions in the reference brain. Klein et al. evaluated 14 methods with four MRI dataset of healthy and young subjects, and showed that these methods perform well in warping the sub‐cortical regions (average DSC above 80%), but even the top performing method ANTs (Avants et al., 2008) and recently‐developed deep learning methods generally have poor performance in warping cortical regions (average DSC between 60% and 70%) (Balakrishnan et al., 2019; Klein et al., 2009; Yang et al., 2017). It is because almost all of the widely used brain image registration techniques that work in the 3D Euclidean space, whether volume‐based, or surface and volume hybrid methods, are based on solving the optimization problem of matching the whole brain at once and suffer from the local minimum problem, resulting in poor registration of brain cortical regions. Recent advances in intensity‐based brain image registration methods used both T1 and T2 weighted images to guide inter‐subject registration (Simonovsky et al., 2016). This multi‐modal approach will help the delineation and matching of gray matter boundaries. However, intensity‐based methods still have the issue of the homogeneous intensities between cortical regions within the cortical gray matter, which hinders the matching of corresponding cortical regions between subjects. Furthermore, many existing retrospective datasets have limited access to scans from other modalities (only T1‐weighted structural scans). Spatial normalization is even more challenging to studies of populations with severe brain morphology changes. For example, caution should be taken when studying aging population, as it has been shown that brain morphology changes along normal aging with grey matter volume reduction (Good et al., 2001), especially in prefrontal regions (Tisserand & Jolles, 2003) and in the medial temporal lobe (Jack et al., 1997). In these cases, inaccurate spatial normalization can transfer population‐related residuals to the normalized group‐level brain activation, which will no longer be a valid representation of the population. Hence, any conclusion drawn with this biased representation will heavily be confounded by the residuals. For example, the age‐related atrophy in the brain of the older participants has shown to further deteriorate the accuracy of the spatial normalization, and subsequently interpreted as age‐related attenuation of BOLD response amplitude (Liu et al., 2017).

We have evaluated our proposed solution using three different experiments: (1) Using simulated 2D images of a single gyrus we demonstrated that our solution not only aligns cortical folding patterns, but also keeps an accurate correspondence in its internal regional structures. Furthermore, we showed using the simulated images that our solution is more robust to the local minimum problem compared to a top performing volumetric registration method ANTs. The simulated experiments highlighted the issues with intensity‐based whole‐brain registration methods (e.g., ANTs) even in a simplified 2D scenario. Whereas, our proposed LG‐RBSN solution addressed these challenges by leveraging the features of using landmark guidance and region‐based registration. However, it should be noted that these simulated experiments cannot substitute the complexities in the registration of real brain images, as human brains have highly convoluted folding patterns and large inter‐subject shape variability. Thus, in this study, we also validated our method with real structural and functional human brain MRI data. (2) Using structural images of human brains, we showed that our solution increases the correspondence between cortical regions, sub‐cortical regions, and cerebral white matter in comparison to the existing top performing volumetric registration method ANTs and a hybrid registration method CVS. In Figure 8, all methods performed better in the registration of sub‐cortical regions than cortical regions, which is expected as sub‐cortical regions are bounded by the white matter and have less complex shapes, rendering an easier case for most of the spatial normalization methods. However, only the method proposed in this study performed well in cortical regions, which have highly convoluted patterns of sulci and gyri, showing a compelling case for the proposed LG‐RBSN method. Our solution also showed that the number of nonpositive Jacobian voxels can be decreased with the utilized residual compensation iterations to the level of ANTs, and is much lower than that of CVS. (3) Using functional images of human brains, we first showed that improving the correspondence between regional structure not only increases the statistical power in detecting smaller activations, but also keeps the false‐positive rate low. This was measured by AUC of the ROC curves, indicating about 6.3% improvement compared to ANTs, and about 1.1% improvement compared to CVS. However, the improvement in the regional correspondence did not transform to the increase in the sensitivity and specificity in the group level statistics when comparing LG‐RBSN and CVS for the right ear auditory stimuli condition. Together, our findings suggest that LG‐RBSN solution is a more accurate and reliable substitute for conventional spatial normalization techniques commonly used in the field.

It is already known that the functional architecture of the brain does not necessarily and accurately follow brain gyri and sulci morphology. Therefore, one might conclude that improving the correspondence between brain structural features (sulci and gyri) might not necessarily translate to improvement in functional correspondence of the aligned regions, and would alter the effects of the improved spatial normalization methods in functional imaging of the brain. Since we currently do not have an accurate measurement of the deviation of the functional architecture from brain morphology, it is difficult to assess any limit in which improvement of the regional correspondence becomes unattainable. And as compared to CVS, the worse performance of LG‐RBSN in the functional evaluation with right ear auditory stimuli condition might be due to this reason. Nonetheless, we have shown that increasing the regional correspondence to 86% still increases the functional correspondence for the visual stimuli and the left ear auditory stimuli conditions, and indicates that we are still operating under such limitation in these conditions. To further align the functional architecture of the brain, in future work, the proposed LG‐RBSN can also be modified to identify landmarks from functional or other imaging modality data, so that landmarks correspondence is established using functional cortical registration methods to help align the brain functional organizations across subjects.

We have preliminarily used region‐based registration methods to align MRI brain images with optimization running separately for each individual brain regional mask instead of the whole brain (Razlighi, 2016). This region‐based spatial normalization method resulted in a 44% improvement of the correspondence between cortical regions (DSC around 0.75) in comparison to the top performing nonlinear whole brain registration (ANTs). However, the inter‐subject variability of cortical regions was still causing the optimization process to fall into local minimums especially in regions with sever age‐related atrophy and deformations, like temporal lobe regions. Additionally, in our previous method, separately warping each region individually with its own warping field can introduce gaps and overlaps in‐between regions. Edwards et al. has also compartmentalized the medical images of the human body into two separate segments in which the rigid and deformable structures of the body were registered independently to their counterparts, transformed separately, and then combined (Edwards et al., 1995). This method improved registration accuracy but was limited for the resultant discontinuity in structural boundaries. This limitation was addressed in a following article (Little et al., 1997) using a single global smooth transformation. The global transformation was composited using a modified radial basis function and the inverse distance interpolation (Shepard, 1968) based on rigid structures within the image. Another similar method used only affine transformations for different segments of the brain (Pitiot et al., 2003), where, each gyrus locally registered to its counterpart using affine transformation for 2D registration of myelin‐stained histological sections of the human brain. Local registration methods interpolate locally linear transformation fields, whereas our method not only uses topographical landmarks to guide the nonlinear registration, but also deals with the regional transition by an IDW interpolation method with enforced bijectivity to ensure both regional and final global warping field are diffeomorphic and topology preserving deformations.

The tradeoff between regional correspondence and bijectivity regularization (for topology preserving deformation) can be tuned to find a balance between the matching of brain structures and number of voxels violating the diffeomorphism. In LG‐RBSN, by tuning the weights between matching and regularization, the number of nonpositive Jacobian voxels can be lowered even to reach zero, however, this will cause a decrease in the correspondence between the cortical regions (the average DSC of cortical region will drop to around 80%). In our experiments, we choose to tolerate 1300 number of voxels (0.0077% of total number of voxels) with nonpositive Jacobian determinant, to achieve about DSC = 86% correspondence between cortical regions, which was the optimal setting in this work, however other applications may require further adjustment to obtain their optimal ranges. In addition, in the IDW interpolation method we set μ=4, which produced acceptable results in our experiments. However, a thorough optimization is required in the future to obtain the optimal value for the μ in each registration application.

In our experiments and results section, the reported DSC for ANTs in this work may seem lower than the ones reported in the literature (Klein et al., 2009). This is because; (a) healthy elderly adults comprise more than one‐third of our sample and generally show excessive brain atrophy in comparison to younger subjects, particularly in the prefrontal and temporal cortical regions (Jack et al., 1997; Tisserand & Jolles, 2003). It has been shown that brain atrophy can significantly alter the effectiveness of brain registration accuracy (Avants et al., 2008). (b) we have evaluated a subject‐to‐template registration while most of the evaluation in the existing evaluation are done with subject‐to‐subject registration (Klein et al., 2009). This will be even more problematic when we have older population in our sample group. In future studies, cerebral cortex regions overlap can be further improved with a custom group average template specifically designed for use with LG‐RBSN.

In our experiments, we evaluated our method with both structural (T1‐weighted structural scan) and functional data (visual and auditory task‐based fMRI scans) from human brain, and we detected statistically significant improvement and stronger brain activations by using our proposed method. This improvement might reduce the required number of subjects to detect the group effects in functional imaging studies. However, future studies are needed to evaluate the robustness of our method by using a large number of subjects. Furthermore, in this study, the samples do not include subjects in the middle‐age group. We highlighted the challenges of dealing with subjects in the old group by using a conventional method, and the superior performance of using our method. We expect that our method will also work well with subjects from the middle‐age group, as these subjects have been shown to have a less age‐related morphological alterations in brain structures compared to subjects in the old group (Razlighi et al., 2017). Future studies are needed to test our method on subjects in the middle‐age group. And it is an important topic to investigate the age‐related brain variability, however, we feel it is out of the scope of this study, as the spatial normalization methods focused on quantifying the inter‐subject brain variability. Our proposed method utilizes the morphometric procedures from FreeSurfer, which have been demonstrated to be insensitive to the heterogeneity in the data acquired from different scanner platforms (Han et al., 2006; Han & Fischl, 2007; Reuter et al., 2012). Therefore, we feel confident that our method will have a comparable performance dealing with brain images acquired from different scanners. However, future work is warranted to test the reliability of our proposed spatial normalization method with multi‐scanner imaging data.

Finally, it would be interesting to evaluate the performance of the LG‐RBSN solution for multivariate techniques such as group independent component analysis (ICA), or partial least‐squares (PLS). The difference between the evaluation of the multivariate and univariate (voxel‐based) methods is that multivariate techniques often require warping the actual 4D fMRI data, whereas univariate analysis can be performed after the first‐level statistical analysis. Therefore, one might expect to obtain a different performance in applying LG‐RBSN to the multivariate data. Furthermore, we only evaluated the LG‐RBSN technique on structural and functional MRI scans. It would be interesting to assess the effectiveness of this method on other MRI modalities such as diffusion weighted imaging, arterial spin labeling, and susceptibility weighted imaging amongst others. We also expect that our proposed spatial normalization method could be extended to enhance spatial normalization accuracy in the other imaging modalities such as positron emission tomography and computed tomography.

## 5 CONCLUSION

Using automatic landmark detection and matching, we have developed and implemented a novel 3D volumetric spatial normalization solution that not only aligns the cortical folding patterns of the brain, but also results in a high correspondence between different regions along the cortical ribbon and their counterparts in the template image. Our solution substantially outperforms the existing top performing volumetric spatial normalization method by giving a significantly higher correspondence between the structure of the neuroanatomical regions, and also yields higher sensitivity and specificity in the group‐level statistics when analyzing task‐based fMRI data with both auditory and visual stimuli. The limited accuracy of conventional methods becomes more prominent when applied to clinical and aging populations with severe alterations in brain morphology. When compared to the healthy group, this population‐based bias has been shown to generate false‐positive findings that have been reported as a genuine breakthrough in the literature (Liu et al., 2017). We conclude that our proposed LG‐RBSN solution is a suitable substitute for the conventional volumetric whole brain registration methods that often fail to generate an accurate correspondence between regions of the cerebral cortex, particularly for clinical and aging populations.

## CONFLICT OF INTEREST

The authors declare no potential conflict of interest.

## Data Availability

The datasets in the current study are available from the corresponding author on reasonable request.
